# A framework to find the logic backbone of a biological network

**DOI:** 10.1186/s12918-017-0482-5

**Published:** 2017-12-06

**Authors:** Parul Maheshwari, Réka Albert

**Affiliations:** 1 Department of Physics, The Pennsylvania State University, University Park, 16802 PA USA; 2 Department of Biology, The Pennsylvania State University, University Park, 16802 PA USA

**Keywords:** Boolean networks, Biological networks, Regulatory functions, Signal transduction network, Attractor, Stable motif, Network model, Sufficient and necessary conditions

## Abstract

**Background:**

Cellular behaviors are governed by interaction networks among biomolecules, for example gene regulatory and signal transduction networks. An often used dynamic modeling framework for these networks, Boolean modeling, can obtain their attractors (which correspond to cell types and behaviors) and their trajectories from an initial state (e.g. a resting state) to the attractors, for example in response to an external signal. The existing methods however do not elucidate the causal relationships between distant nodes in the network.

**Results:**

In this work, we propose a simple logic framework, based on categorizing causal relationships as sufficient or necessary, as a complement to Boolean networks. We identify and explore the properties of complex subnetworks that are distillable into a single logic relationship. We also identify cyclic subnetworks that ensure the stabilization of the state of participating nodes regardless of the rest of the network. We identify the logic backbone of biomolecular networks, consisting of external signals, self-sustaining cyclic subnetworks (stable motifs), and output nodes. Furthermore, we use the logic framework to identify crucial nodes whose override can drive the system from one steady state to another. We apply these techniques to two biological networks: the epithelial-to-mesenchymal transition network corresponding to a developmental process exploited in tumor invasion, and the network of abscisic acid induced stomatal closure in plants. We find interesting subnetworks with logical implications in these networks. Using these subgraphs and motifs, we efficiently reduce both networks to succinct backbone structures.

**Conclusions:**

The logic representation identifies the causal relationships between distant nodes and subnetworks. This knowledge can form the basis of network control or used in the reverse engineering of networks.

**Electronic supplementary material:**

The online version of this article (doi:10.1186/s12918-017-0482-5) contains supplementary material, which is available to authorized users.

## Background

Dynamics at the cellular level are governed by various interaction networks among biomolecules, including signal transduction, metabolic and gene regulatory networks. The nodes of these networks are different types of biomolecules: proteins, mRNAs and small molecules, while the edges signify biochemical reactions, protein-protein interactions or transcriptional regulation via directional mass or information flow. Many cellular functions involve numerous nodes and interactions, hence these networks are large and complex. Various modeling techniques, involving different levels of detail and necessitating knowledge of varying amounts of biological information, have been developed to analyze these networks [1–14]. A lot of quantitative information can be obtained from continuous models formulated as differential equations, construction of which needs knowledge of the values of chemical kinetic parameters, reaction rates, etc [3–6]. In contrast, discrete dynamic models give a good qualitative understanding of the system using only the inhibiting or activating nature of the interactions without the use of any kinetic parameters [7–14]. The simplest discrete dynamic framework, called a Boolean model, characterizes each node with two states: 1 (ON, meaning above-threshold expression or activity) or 0 (OFF, meaning below-threshold expression or activity) [15–18]. In this model, the future state of a node is determined by a logic function of the current states of its regulators. This logic function is called a Boolean regulatory function and usually written as *f*_*Node*_. The network dynamics can be understood by starting from a suitable initial state and successively evaluating the Boolean regulatory function of each node to find its next state. The resulting system trajectories can be summarized as a state transition graph whose nodes are states of the system and whose edges represent state transitions [18, 19]. If a state in this state transition graph does not have an outgoing edge to another state, it is a fixed point (also called steady state). If the transition graph contains a cycle or a strongly connected component of certain states, and if there are no transitions that go out of this strongly connected component, the respective states form a complex attractor. Often, Boolean networks are too large and complex to be analyzed by exhaustive simulations of the system trajectories. An efficient alternative is to identify so-called stable motifs [20], generalized positive feedback loops which when stabilized in a certain state maintain that fixed state of the constituent nodes. Successive identification and reduction of stable motifs can be used to identify the steady states of the system, or the partial steady states wherein a fraction of the nodes stabilize and the remaining nodes may oscillate [20–22]. Similarly, oscillating motifs, i.e., feedback loops where the constituent nodes’ states oscillate, represent complex attractors of the network [23]. The succession diagram resulting from the iterative identification and elimination of stable motifs reflects the points of no return in the system’s dynamics and can be used as an alternative of the state transition graph [20].

To complement these analyses of the network’s dynamics and attractors, we propose a simple logic framework for a deeper insight into the causal structures that ultimately determine the system dynamics. This paper is organized in two major parts, where the first part builds up the theoretical formalism and the second part contains two applications to published Boolean models. We start with describing the proposed causal edge representation and the corresponding logic implications. Once the edges are labeled with causal logic, we identify ways to find paths and subnetworks that, in their completeness, imply a particular logic. This is a rather strong and useful implication. For example, if there is a node X at a significant graph-theoretic distance from another node Y, and X has a logical implication on Y, we can predict the state of Y if we know the state of node X without the knowledge of the states of any other nodes of the network. The logic subgraphs and paths have interesting properties and can be used to develop an efficient network reduction technique. We also identify feedback loops with logic implications that are equivalent to stable motifs. We then develop a succinct representation of the relationship between input signals, the motifs and output (or sink) nodes. This representation intuitively expresses the network logic and indicates which nodes can drive the network to a particular steady state.

Using the causal logic framework, we analyze two intracellular networks, one from humans and the other from plants. The first network characterizes the epithelial-to-mesenchymal transition (EMT), which is a developmental process exploited by cancer cells to initiate metastasis and tumor invasion. We summarized this 69 node EMT network and its dynamics into a succinct 22 node backbone graph. The second network corresponds to the opening and closing of stomatal pores, which is critical for the regulation of carbon-dioxide intake and water vapor loss. In drought conditions, the phytohormone abscisic acid (ABA) acts as a signal in an intracellular network that induces stomatal closure for prevention of water loss. We summarized the 80 node ABA-induced stomatal closure network to a 14 node backbone structure.

## Methods

**Fig. 1:**
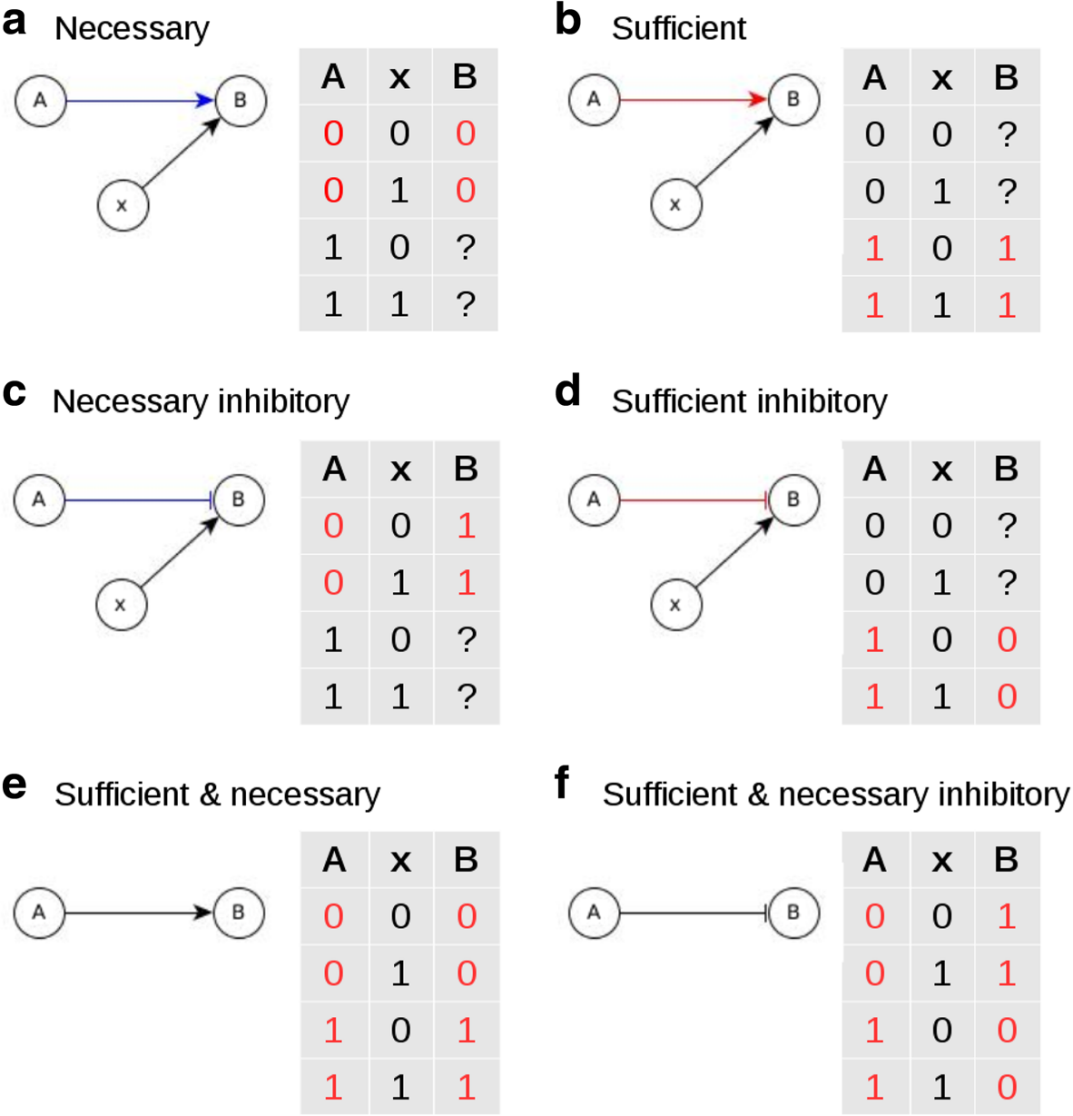
Causal relationship between two nodes expressed by the edge type. Edges ending in an arrow (→) signify activation and edges ending in a bar (⊣) signify inhibition; node *x* signifies other regulator(s) of *B*. **a** *A* is necessary for *B*, meaning that whenever *A* is OFF, *B* must be OFF, regardless of the state of *x*; **b** *A* is sufficient for *B*, which means that *A* being ON implies that *B* is ON, regardless of the state of *x*; **c** *A* is a necessary inhibitor of *B*, i.e., *A* must be ON to inhibit *B*, implying that when *A* is OFF, *B* must be ON, regardless of the state of *x*; **d** *A* is sufficient to inhibit *B*, i.e., whenever *A* is ON, *B* must be OFF, regardless of the state of *x*; **e** *A* is sufficient and necessary for *B*, i.e., *B* is always stabilized in the same state as *A*; **f** *A* is a sufficient and necessary inhibitor for *B*, i.e., *B* is always stabilized in the state opposite of that of *A*. Blue edges represent necessary relationships, red edges represent sufficient relationships and black edges represent a sufficient and necessary relationship i.e., when the target node has only one regulator. The corresponding truth table of steady states for each edge type is on the right. States not specified by the logic relationship between *A* and *B*, which therefore depend on *x*, are shown as question marks. The states of *A* shown in red are the causative states and the states of *B* shown in red are the resultant states

The structure of a regulatory network expresses the relationship between two nodes (e.g. between a kinase and its target protein) but the logical implication of the relationship is not completely specified; all we know from the edge between the two nodes is whether the activity of the source node influences that of the target node either in an activating or in an inhibiting manner. The Boolean regulatory function of a target node describes the combined effect of all of its regulators and does not separate the effect of a single regulator. We propose a framework to also express the individual logic effect of a regulator node on a target node as a property of the edge between them and hence communicate the exact relationship between the regulator and the target node through the network representation. We characterize edges as sufficient or necessary, which tells whether the activity of the regulator node is sufficient or necessary to activate (or de-activate) the target. This means that if we know the state of a regulator, depending on the edge type, we can have a definite knowledge of the state of the target node irrespective of the state of the rest of the network, as illustrated in Fig. 1. This representation incorporates the edge logic into the network structure. For consistency, we consider the case of sustained (in)activity of the regulator node and focus on the long-term steady state of the target node. Alternative situations are considered in the “Discussion” section. For an activating regulator *A* that has a necessary edge to a target node *B*, deactivating the regulator (fixing it in the OFF state) ensures that the target is deactivated irrespective of the rest of the network, as shown in Fig. 1a. The Boolean regulatory function for this target would include an AND clause with this regulator. For example, in Fig. 1a, *f*_*B*_=*A* AND *x*. On the other hand, if an activating regulator node *A* is sufficient for a target node *B*, it means that if *A* is ON, then *B* will also stabilize to ON, irrespective of the state of the rest of the network, as shown in Fig. 1b. Consequently, in the Boolean regulatory function of *B*, *A* is connected by an OR operator to the other regulator(s) of *B*, i.e. *f*_*B*_=*A* OR *x*. To understand how this framework realizes inhibitory edges, it is important to note that inhibition implies the capability to deactivate something. A necessary inhibitory edge, e.g., *A*⊣*B* in Fig. 1c, means that the sustained activity of *A* is necessary to inhibit the target node *B*, that is, if the regulator node is kept inactive (*A*=OFF), then the target node would be activated (*B*=ON) irrespective of the states of other regulators. This is observed when the Boolean regulatory function of the target contains the OR NOT clause with this regulator, i.e., *f*_*B*_=(NOT *A*) OR *x*. A sufficient inhibitory edge, e.g., *A*⊣*B* in Fig. 1d, means that sustained activity of the regulator node (*A*) is sufficient to deactivate the target node (fixes *B*=OFF) irrespective of the state of the rest of the network. The Boolean regulatory function of *B* must hence contain the AND NOT clause with the regulator, i.e., *f*_*B*_=(NOT *A*) AND *x*. If an edge is both sufficient and necessary then the source node is the only regulator of the target node (Fig. 1e), so the Boolean regulatory function for *B* is: *f*_*B*_=*A*. When the target node has just one regulator that is inhibitory, there is a sufficient and necessary inhibiting edge as in Fig. 1f. The Boolean regulatory function in this case is *f*_*B*_=NOT *A*.

**Fig. 2:**
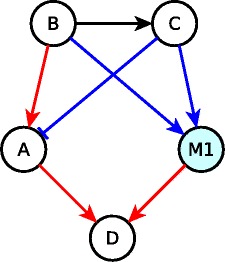
Example of a causal edge representation of a Boolean network. Blue edges represent necessary relationships, red edges represent sufficient relationships and the black edge represents a sufficient and necessary relationship. Node *M*1 (blue) is a mediator node. Node *B* is a signal (source node) while node *D* is an output node. The Boolean rules are: *f*_*A*_=*B* OR (NOT *C*); *f*_*C*_=*B*; *f*_*D*_=*A* OR (*B* AND *C*)

A target node can only have certain combinations of logical regulators. For example, if a regulator is directly (through a single edge) sufficient for a target node, there cannot be another regulator (independent of the first) that is directly necessary for this target since the case where the necessary regulator is OFF while the sufficient regulator is ON yields contradictory specifications for the target node. Sufficient regulators are only compatible with necessary inhibitors (i.e. resulting in Boolean regulatory functions of the type “ *A* OR NOT *B*”), and necessary regulators can only be combined with sufficient inhibitors (in Boolean regulatory functions of the type “ *A* AND NOT *B*”). There can be cases where a regulator is neither directly sufficient nor necessary for the target but it has a logical implication when combined with other regulators. For example, consider the following Boolean regulatory function: *f*_*D*_=*A* OR (*B* AND *C*). Here, *A* is sufficient for *D* while *B* is neither sufficient nor necessary for *D*. The combination (*B* AND *C*) is sufficient for *D*. We introduce mediator nodes in the network to represent such cases of relationships embodied by groups of nodes. The above example is shown in Fig. 2 where *M*1 is a mediator node and *f*_*M*1_=*B* AND *C*. This implies that *B* is necessary for *M*1 while *M*1 is sufficient for *D* with the equivalent Boolean regulatory function: *f*_*D*_=*A* OR *M*1. The use of mediator nodes ensures that all activating edges incident on a node (or a mediator node) have the same color (i.e. logic implication) while all inhibiting edges incident on the same node are of the opposite color of activating edges. For simplicity and optimization of this representation, we propose that the Boolean regulatory functions be expressed either in Conjunctive Normal Form (CNF) or in Disjunctive Normal Form (DNF). In this work, we chose the form (CNF or DNF) that minimizes the number of edges and mediator nodes. We ensured that the chosen form of the Boolean regulatory function covers all prime implicants.

**Fig. 3:**
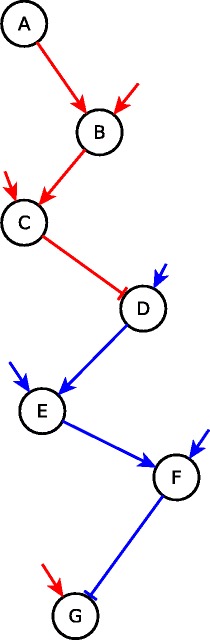
Possible logical linear paths. *ABC* is sufficient; *ABCD* is sufficient inhibitory; *DEF* is necessary; *DEFG* is necessary inhibitory; *ABCDEFG* is sufficient. Incoming arrows that have no starting points indicate unknown additional regulators

Two consecutive edges can lead to a particular logical implication (e.g. sufficient), thus defining the *chaining of edges*. For example, two sufficient edges, such as *A*→*B* and *B*→*C* in Fig. 3, can be chained to yield a sufficient relationship, i.e., *A* is sufficient for *C*. The *B*→*C* sufficient edge can be chained with the sufficient inhibitory edge *C*⊣*D* and yields a sufficient inhibitory relationship, meaning that setting *B* ON stabilizes *D* to OFF irrespective of the states of all nodes other than *B*, *C* & *D*. By extension, a two-edge path (e.g. *ABC*) may be chained with an edge to yield a logic implication of the resulting three-edge path (e.g. the *ABCD* path is sufficient inhibitory). If a linear path (succession of edges) has all its edges chaining in a certain manner, then the path can be attributed a particular logical implication (e.g., sufficient, necessary). For example, if each edge in a linear path in the network is sufficient (respectively, necessary) then the path is also sufficient (respectively, necessary), see Fig. 3. We determined all the cases of chaining of consecutive edges or paths and expressed it in a *chain function*, summarized in Table 1. With the help of this function, we can determine the causal effect of a distant node on another. For example, if a linear path is sufficient and we fix the first node to ON, then the last node will also stabilize to the ON state. As an additional example, if a sufficient inhibitory path (e.g. *ABCD* in Fig. 3) is followed by a necessary path (*DEF*), fixing the first node to ON would stabilize the target of the sufficient inhibitory path (*D*) to OFF and since this target is the first node of the necessary path, it will stabilize the last node of the combined path (*F*) to OFF. This combination would hence result in the same effect as a sufficient inhibitory path, as shown in Table 1.

**Table 1:** The chain function: the rows represent the logic type of the preceding edge or path and the columns represent the type of the succeeding edge or path; each cell indicates the type of the resulting path; “-” indicates that there is no particular logical implication when such paths are chained

	Succeeding	suff	necc	suff & necc	suff inh	necc inh	suff & necc inh
Preceding							
suff		suff	-	suff	suff inh	-	suff inh
necc		-	necc	necc	-	necc inh	necc inh
suff & necc		suff	necc	suff & necc	suff inh	necc inh	suff & necc inh
suff inh		-	suff inh	suff inh	-	suff	suff
necc inh		necc inh	-	necc inh	necc	-	necc
suff & necc inh		necc inh	suff inh	suff & necc inh	necc	suff	suff & necc

“suff” stands for sufficient, “necc” for necessary, “suff & necc” for sufficient and necessary, i.e. only one regulator (activating), “suff inh” stands for sufficient inhibitory, “necc inh” for necessary inhibitory and “suff & necc inh” for sufficient and necessary inhibitory, i.e., only one regulator (inhibiting)

## Results

### Subgraphs with causal logic implication

If there is no linear path of a particular causal implication between a pair of nodes, there can still be a logic relationship between them via the combination of multiple paths. These combinations of multiple paths between a pair of nodes, which we will refer to as subgraphs, can be used to understand what can be achieved by fixing the source (starting) node to a particular state. For example, having a necessary subgraph implies that if we fix the source (starting) node of the subgraph to OFF then the target (last) node of the subgraph stabilizes in the OFF state irrespective of the state of the rest of the network (i.e. of the nodes not contained in the subgraph).

**Fig. 4:**
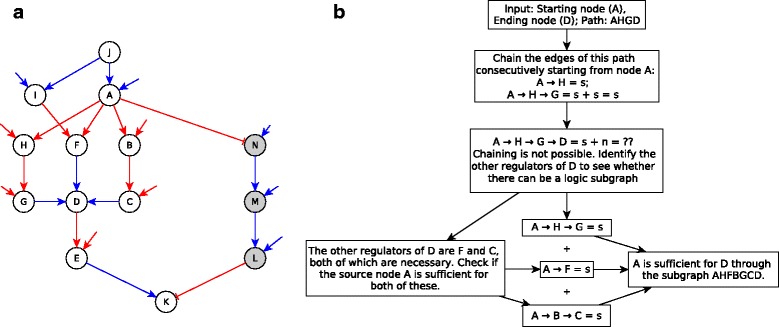
Sufficient and necessary subgraphs. **a** *JIAF* is a necessary subgraph where *I* and *A* are sufficient regulators of the subgraph’s target, *F*, and the subgraph’s source *J* is necessary for each of these. *AHFBGCD* is a sufficient subgraph where *G*, *F* and *C* are the necessary regulators of the target node *D* and the source node *A* is sufficient for each of these regulators. *AHFBGDCNMELK* is a sufficient subgraph where *E* is a necessary regulator and *L* is a sufficient inhibitory regulator of the target node *K* and the source *A* is sufficient for *E* while it is sufficient inhibitory for *L*. **b** Flowchart illustrating the application of the algorithm to identify the sufficient subgraph AHFBGCD from the network in panel A. The abbreviations used for causal logic implications are as follows: s-sufficient; n-necessary; si-sufficient inhibitor

We find that a sufficient subgraph is formed if there are multiple necessary regulators (and no other types of regulators) of the target node (e.g. *G*, *F* and *C* in Fig. 4 are necessary regulators of *D*) and the source is sufficient for each of these necessary regulators. The source may be sufficient for each of the necessary regulators via an edge, a path (as is *A* for nodes *G*, *F* and *C* in Fig. 4), or even a subgraph. Another possible architecture of a sufficient subgraph is when there are multiple sufficient inhibitory regulators (and no other types of regulators) and the source is a sufficient inhibitor of each of these regulators. In this case, keeping the source ON stabilizes all the regulators in the OFF state, allowing the target to turn ON. Finally, these two types of relationships can be mixed: if the target node has necessary and sufficient inhibitory regulators (and no other regulators), and the source is sufficient for the necessary regulators and a sufficient inhibitor of the sufficient inhibitory regulators, the source is overall sufficient for the target. For example, in the sufficient subgraph from *A* to *K* in Fig. 4 the target node *K* has a necessary regulator, *E*, and a sufficient inhibitory regulator, *L*. The source node *A* is sufficient for *E* via a subgraph from *A* to *D* and then an edge from *D* to *E*; while *A* is a sufficient inhibitor of *L* via a path (one can quickly check from Table 1 that *ANML* is indeed a sufficient inhibitory path). When *A* is fixed to the ON state, the *A*−*E* sufficient subgraph ensures that *E* stabilizes to ON as well, while the *A*−*L* sufficient inhibitory path ensures that *L* stabilizes in the OFF state. Since the Boolean regulatory function of *K* is *f*_*K*_=*E* AND NOT *L*, the target node *K* stabilizes in the ON state, making the *A*−*K* subgraph a sufficient subgraph. Generally, for each logic implication between a source and a target there exist multiple pairings of logic implications that may not be defined for chaining of two paths but can yield the desired logic implication if one of the pairings apply for each regulator of the target. Our previous example showed that the pairing of sufficient with necessary, and the pairing of sufficient inhibitory with sufficient inhibitory, yields a sufficient subgraph. We hence propose a *subgraph chain function*, in analogy to the chain function for paths, and indicate it in Table 2. Figure 4 illustrates sufficient and necessary subgraphs.

**Table 2:** The subgraph chain function: The rows represent the logic type of the preceding edge or path and the columns represent the type of the succeeding edge or path; each cell corresponds to the type of subgraph that may exist if there is a pairing of the preceding relationship with the succeeding relationship

	Succeeding	suff	necc	suff inh	necc inh
Preceding					
suff		-	suff	-	suff inh
necc		necc	-	necc inh	-
suff inh		suff inh	-	suff	-
necc inh		-	necc inh	-	necc

A subgraph exists only if this chain function gives the same result (the same values in the corresponding cells) for all regulators. “-” indicates that there is a path, see Table 1. “suff” stands for sufficient, “necc” for necessary, “suff & necc” for sufficient and necessary, i.e. the only regulator (activating), “suff inh” for sufficient inhibitory, “necc inh” for necessary inhibitory and “suff & necc inh” for sufficient and necessary inhibitory, i.e., the only regulator (inhibiting)

We implemented the path and subgraph chain functions (Table 2) to find subgraphs between pairs of nodes with one of the logical implications (sufficient, necessary, sufficient inhibitor, necessary inhibitor, sufficient and necessary, or sufficient and necessary inhibitor). The algorithm (presented in more detail in Additional File 1) takes as input a network with assigned edge logic implications (which in turn can be constructed from the Boolean rules of each node), a start-node (*S*) and an end-node (*E*). The first step of the algorithm is to create a list of all simple paths from *S* to *E*. This list is obtained using the *networkx.all_simple_paths* built-in function from the Networkx graph library which uses a modified depth-first search to generate the paths. For reducing computation time, the list is sorted by increasing path length. The paths are scanned one by one, starting with the shortest, until we find a subgraph with a logical implication. For each path, the algorithm tries to chain consecutive edges, i.e., obtain the cumulative logical implication of the consecutive edges. During this chaining of edges, if a certain edge (for example *A*→*B*) cannot be chained, i.e., the combination of the path from *S* to *A* chained with the edge *A*→*B* yields “-” in the chain function in Table 1, then the algorithm tries to find a subgraph from *S* to *B* by looking at the logical implication of the start-node *S* on each of the regulators of node *B*. So, the algorithm now scans paths from *S* to other regulators of *B*. If the subgraphs/paths from *S* to each of the regulators of *B* chain with the respective edges between each regulator and *B*, and yield the same subgraph type as per the subgraph chain function in Table 2, the corresponding implication is recorded and the next edge in the path is scanned. If the same subgraph type is not yielded, this means the path being scanned does not have a logical implication hence we scan the next path between *S* and *E*. If while scanning a path, we reach the node *E* (i.e., the path is completely scanned) then the resultant logical implication and the complete subgraph is returned as output. If all the paths have unfinished scans (i.e., none of them are a logic path or subgraph), the function reports no logic relationship between the given pair of nodes. An illustration of the algorithm is provided in panel B of Fig. 4, where the goal is to find the subgraph between node *A* and node *D* of Fig. 4a. In this example, the target node *D* has three necessary regulators: *C*, *F* and *G*, and the source node *A* is sufficient for each of these regulators.

### Properties of logic subgraphs

The causal logic representation allows the identification of the conditions under which a cycle (feedback loop) can intersect a logic path or subgraph. Here, intersection of a cycle and a subgraph means that two or more nodes and at least one edge is shared by the cycle and the subgraph. We find that a sufficient subgraph that does not contain any inhibitory edges cannot intersect a necessary cycle. Analogously, we also find that a necessary subgraph without any inhibitory edges cannot intersect a sufficient cycle.

**Fig. 5:**
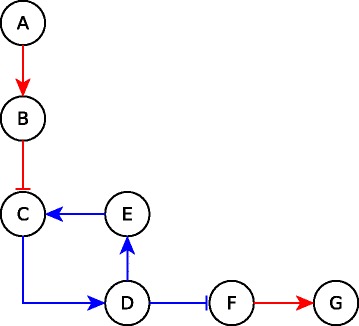
Necessary cycle intersecting with a sufficient subgraph. *ABCDFG* is a sufficient path intersecting the necessary cycle *ECD*. The intersection, i.e. the edge *C*→*D*, follows a sufficient inhibitory portion of the sufficient subgraph (*ABC*)

A necessary cycle could intersect a sufficient subgraph containing inhibitory edges only if the intersection follows a sufficient inhibitory section of the subgraph. An example is illustrated in Fig. 5, where the intersection *CD* of the necessary cycle *CDE* with the subgraph (path) *ABCDFG* follows a sufficient inhibitory section *ABC*. Analogously, a sufficient cycle can intersect a necessary subgraph containing inhibitory edges only if the intersection follows a necessary inhibitory section of the subgraph. A detailed proof for these properties is given in Additional file 2.

We also studied the case of two subgraphs that have different starting (source) nodes but end in the same target node; we will refer to this situation as co-pointing subgraphs. We find that if there are two co-pointing subgraphs, one of which is necessary while the other is sufficient, then they must intersect. If either of the starting points is a signal node (with in-degree 0) then the starting node of the other subgraph must lie in the intersection. If the subgraph starting with a signal node is sufficient, then this signal node must be sufficient for the starting node of the necessary subgraph. This situation occurs frequently when using genetic or pharmacological knockout experiments to identify putative signal transduction mediators. The signal is, or at least it is strongly expected to be, sufficient for a relevant target node that expresses the outcome of the signal transduction process (assuming the presence of molecules generally necessary for life). Finding that this knockout disrupts the signal transduction process and eliminates the outcome even in the presence of the signal makes the putative mediator necessary for the target node. Our result formalizes and proves the implicit conclusion that the knocked-out node is indeed a mediator of the process, i.e. a connection from the signal to the knocked-out node exists.

### Stable motifs

**Fig. 6:**
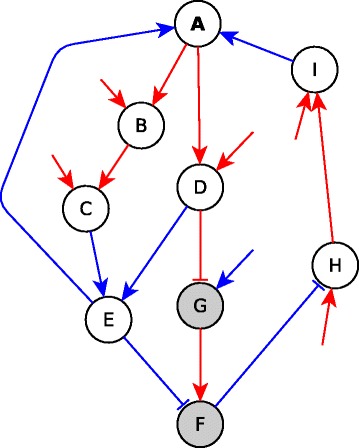
Illustration of a sufficient cyclic subgraph that corresponds to a stable motif. In the *ABCDE* subgraph the target node *E* has two necessary regulators, *C* and *D*, *ABC* is a sufficient path and *A*→*D* is a sufficient edge, making the entire subgraph sufficient. In the *ABCDEGF* subgraph the target node *F* has two regulators, *E* and *G*; the *A* to *E* subgraph is sufficient while the *A* to *G* path is sufficient inhibitory. Using the subgraph chaining function at node *E*, we chain the sufficient subgraph *ABCDE* with the necessary inhibitory edge *E*⊣*F*, giving us a sufficient inhibitory relationship (check Table 2). Similarly, at node *G*, we chain the sufficient inhibitory path *ADG* with the sufficient edge *G*→*F* which gives sufficient inhibitory (check Table 1). Since the subgraph chaining for both regulators of the target node *F* gives the same result, we have a sufficient inhibitory subgraph from *A* to *F*. *FHI* is a necessary inhibitory path. Combining subgraph *ABCDEGF* and path *FHI*, we have a sufficient subgraph *ABCDEGFHI* with the source node *A* and target node *I*. Node *A* has two necessary regulators, *E* and *I* and we know that *A* is sufficient for both of these regulators, hence making *A* sufficient for itself. We thus have a sufficient cyclic subgraph (i.e. a sufficient subgraph starting as well as ending at the same node, *A*) which in effect is a stable motif. Nodes with white background are in the ON state, while those with gray background are in the OFF state in the stabilized state of the motif. Node names marked in bold indicate driver nodes

If a subgraph of a particular logic implication forms a cycle, i.e., the source (starting) node and the target (ending) node of the subgraph are the same, we call it a cyclic subgraph. Such cyclic subgraphs may correspond to stable motifs. A stable motif is a strongly connected subnetwork which maintains a fixed state of its constituent nodes regardless of the rest of the network [20]. For a cyclic subgraph to be a stable motif, it should be either sufficient, necessary or sufficient and necessary for one of its nodes when this node is considered as both the source and the target of the subgraph. A stable motif represented by a sufficient cyclic subgraph is illustrated in Fig. 6. A sufficient cyclic subgraph is a stable motif because if the source is activated, there is a path or subgraph from the source to itself, which keeps it activated i.e. maintains its fixed ON state. The starting (and ending) node of the cyclic subgraph, when fixed to the state corresponding to the stable motif, can drive the motif, i.e., stabilize all the nodes in the motif. We refer to these nodes as *driver nodes*. We also find that a given stable motif can have more than one driver node if it corresponds to multiple cyclic subgraphs (same motif but different starting/ending nodes): the starting/ending nodes of each of these cyclic subgraphs give us the set of driver nodes. In addition, there may be a node external of the motif that can fix the state of a driver node of the motif; we refer to such node as *external driver node*.

Owing to the fact that cyclic subgraphs have the same starting node and ending node, they are naturally denser and typically more complex than a simple subgraph of the same size, except for rare cases of self-loops and simple cycles. Hence, the identification of cyclic subgraphs or stable motifs is a “worse-case” runtime of the algorithm. To test the efficiency of our subgraph finding algorithm, we searched for cyclic subgraphs in an ensemble of random Boolean networks (RBNs). An RBN is a network randomly selected with equal likelihood from a set of all possible Boolean networks with a given number of nodes and edges. We analyzed RBNs with 20-60 nodes and in-degree varying between 1.2-2.1 and observed that the runtime was increasing exponentially with the in-degree (for example, for RBNs of 20 nodes, *t*≈10^−9^ *e* *x* *p*(7*k*_*in*_) *s* *e* *c*). Further detail on the analysis of RBNs and runtime complexity plots are presented in Additional file 1.

### Logic-based network reduction

Most complex networks have a large number of nodes and edges, making their dynamic modeling difficult and their state space vast. Network reduction methods are often used to decrease the state space to a manageable size [24]. Albert et al. in [25] devised two methods for network reduction and showed their utility in constructing a sparse equivalent network consistent with a set of indirect experimental observations. The two methods are Binary transitive reduction (BTR) and Pseudo-Vertex Collapse (PVC) [26]. Binary transitive reduction (BTR) is the removal of an edge between a pair of nodes if a path of the same direction and sign exists. Edges known to correspond to interactions, referred to as “critical” edges, are never removed in the reduction process. As a tool to reduce the number of nodes in a network, while maintaining all the relationships between important nodes, certain less important nodes (vertices) of the network are marked as pseudo-vertices. In PVC, if two pseudo-vertices have the exact same set of in- and out-neighbors with the corresponding signs (promoting or inhibiting) being equal, then the two pseudo-vertices are collapsed to generate a new pseudo-vertex with the neighbor set of either of the two. PVC and BTR can be iteratively used to reduce a larger and denser network.

In this work, we propose the logic-preserving versions of BTR and PVC, which minimize the loss of information incurred in the reduction process. We define logical binary transitive reduction (*l*-BTR) wherein an edge is deleted if there exists an alternate path with the same direction, sign, and logical implication as the edge. In *l*-BTR as well, we fix a set of critical edges.

In the logic preserving version of PVC, i.e., logical vertex collapse (LVC), we collapse two vertices if they have the same neighbor set with equal corresponding directions, signs and logical implication (edge color). We note that instead of designating less important nodes as pseudo-vertices, we define a set of critical nodes and permit the collapse of all other nodes with each other or with critical nodes. Thus, *l*-BTR and LVC are stronger logical versions of BTR and PVC respectively. Iterative reduction using these two methods gives a network with the same causal logic information and hence closer to the entirety of experimental evidence. In addition to the above two methods for reduction, we also use edge collapse (i.e., collapsing two nodes into one) in case the source node of the edge is the only regulator of the target node. If a node is an only regulator of the target, the edge between the two is sufficient and necessary and hence the Boolean regulatory function is: *f*_*target*_=*r* *e* *g* *u* *l* *a* *t* *o* *r*. Since this work deals with steady state analysis, the elimination of a time delay does not have any negative implications, so we collapse the regulator and the target to generate a new node *w* such that all the incoming edges into the regulator are set as incoming into *w* while all the outgoing edges from the target are set as outgoing from *w*. As illustrated in the “Application I: logic subgraphs, stable motifs and logic backbone of the EMT network” section, these three methods are greatly useful in reducing a large and dense network while keeping its logic properties intact.

### The logic backbone structure

In Boolean models of gene regulatory networks, stable motifs offer a great insight into the system’s dynamic repertoire and trajectories [20, 21]. Using the causal logic representation, we propose the construction of a logic backbone structure for the Boolean network, which expresses the network in a very condensed form and gives an intuitive idea of the system trajectories. This logic backbone, comprised of the signal(s), the stable motifs (cyclic subgraphs) and the output(s), is a network expressing the logic relationships between these elements. Specifically, a sufficient edge from a signal to a motif means that the sustained presence of the signal can stabilize the motif. A sufficient edge from a stable motif to an output means that the stabilization of the stable motif into its associated state leads to the stabilization of the output in the ON state. A logic backbone structure hence gives us an understanding of which components of the network (signals or motifs) can control which other components. The edges between different elements in the backbone are a representation of subgraphs or paths between them. We present the logic backbones of two specific network models in the “Application I: logic subgraphs, stable motifs and logic backbone of the EMT network” and “Application II: Logic subgraphs and logic backbone of the ABA network” sections.

If a particular stable motif can drive the output or the entire network to a steady state of biological importance, then the driver nodes (and external driver nodes) of that motif become crucial. These nodes act as control nodes and a state change in one of them due to, for example, a mutation could stabilize the entire network to a fixed state. If a system is known to incur state-changing mutations, we can estimate the probability that a mutation’s effects will propagate by knowing the fraction of nodes that are driver nodes. So, for a network with *n* nodes, of which *n*_*d*_ are driver nodes of motifs that can drive the system to the attractor A, then a random state-changing mutation will drive the system to the attractor A with probability *n*_*d*_/*n* and the effects of the mutation will stay localized with probability 1−*n*_*d*_/*n*.

### Application I: logic subgraphs, stable motifs and logic backbone of the EMT network

Epithelial-to-mesenchymal transition (EMT) is a developmental process which is exploited by cancer cells to initiate metastasis and tumor invasion [27, 28]. Steinway et al. constructed a signal transduction network and Boolean model of this transition in response to more than 10 signals including transforming growth factor beta (TGF *β*), platelet-derived growth factor (PDGF) and Sonic hedgehog (SHH). An important marker of the epithelial to mesenchymal transition is loss of E-cadherin expression, which is known to be mediated by seven transcription factors which are reported by Steinway *et al* as SNAI1, SNAI2, ZEB1, ZEB2, HEY1, FOXC2 and TWIST1 [21]. Steinway et al. include an output node, EMT, whose sole and negative regulator is E-cadherin. The Boolean model indicates that the system has two steady state attractors, one almost the opposite of the other: one corresponding to the epithelial state, when the output node is in the OFF state, and one corresponding to the mesenchymal state. Steinway et al. found that this network has eight stable motifs (i.e., generalized feedback loops) and the stabilization of any one of those can lead to steady states that correspond to the mesenchymal state.

**Fig. 7:**
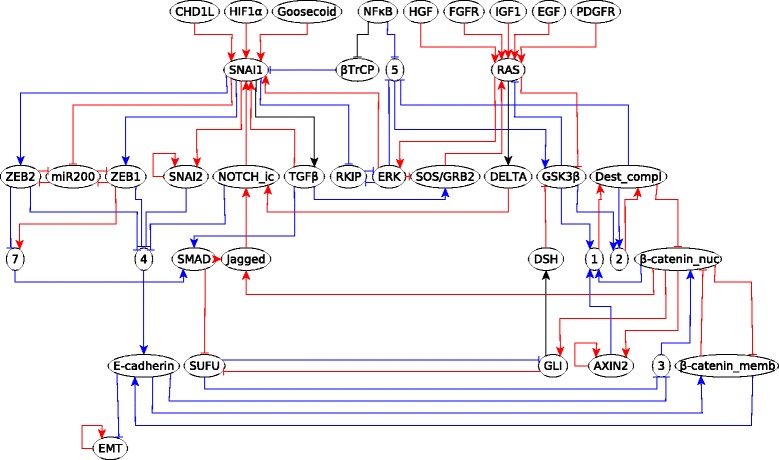
Reduced EMT network in the causal logic representation. Red edges are sufficient, blue edges are necessary and black means that the source node is the only regulator of the target node. All the nodes with numbers (1-10) as labels are mediator nodes. The full names of the abbreviated node names are given in Table 3 and in Supplemental Table 1 of [21]

**Fig. 8:**
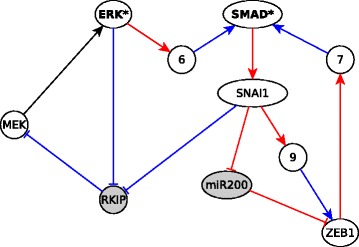
Extended cyclic subgraph with two collective driver nodes corresponding to a stable motif. There is a sufficient subgraph starting from SMAD and ERK collectively and ending at them. Hence, SMAD and ERK form a set of collective driver nodes. Nodes with white background are in the ON state, while those with gray background are in the OFF state in the stabilized state of the motif. Node names marked in bold and starred refer to collective driver nodes

**Fig. 9:**
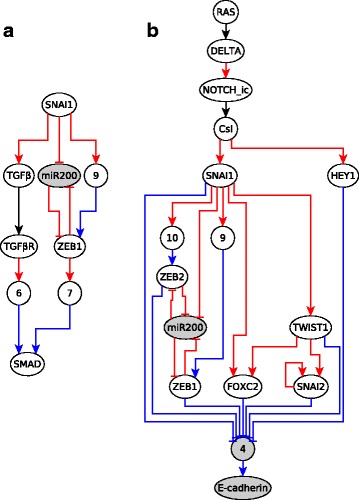
Interesting subgraphs from the EMT network. **a** Sufficient subgraph from SNAI1 to SMAD (SNAI=ON => SMAD=ON); **b** Sufficient inhibitory subgraph from RAS to E-cadherin (RAS=ON => E-cadherin=OFF). Nodes with white background go into the ON state, while those with gray background go to the OFF state when the source node is fixed in the ON state

We analyze the EMT network and its features by transforming it to a causal logic representation. We used the network and Boolean regulatory functions constructed from the literature by Steinway et al. [21]. Prior to the analysis we reduced the 69-node EMT network to a 27 node network using logical vertex collapse, logical binary transitive reduction, and edge collapse. The reduced network is expressed in causal logic representation in Fig. 7. We implemented the subgraph finding algorithm (see Additional file 1) to find subgraphs in this network. To find the stable motifs, i.e., the cyclic subgraphs, the algorithm was modified to have the source node of the subgraph coincide with the target node. We found multiple cyclic subgraphs which corresponded to seven of the eight stable motifs reported by Steinway et al. Some of the cyclic subgraphs found were a union of two or more stable motifs. This is because stable motifs were defined in [20] as the smallest self-sustaining subgraphs, to enable their combinatorial composition. We were however missing one stable motif (the one represented in Fig. 8). We found that in addition to stable motifs representable by cyclic subgraphs, there are some stable motifs which cannot be stabilized by the sustained state of just one node but instead need two or more nodes in a fixed state. These stable motifs can be found by identifying subgraphs from and to a group of nodes. In such a subgraph, which we will refer to as *extended cyclic subgraph*, all the starting nodes, which we will call *collective driver nodes*, need to be fixed to a certain state in order to stabilize all the ending nodes to a certain state. For example, the extended cyclic subgraph in Fig. 8 has two collective driver nodes: SMAD and ERK. Extended cyclic subgraphs are explained in detail in Additional file 3. All the stable motifs of the EMT network, corresponding to cyclic subgraphs or extended cyclic subgraphs, are shown in Additional file 4. We find multiple subgraphs, a few of which are shown in Fig. 9. There is a sufficient subgraph from SNAI1 to SMAD, depicted in Fig. 9a. Since there is a sufficient edge from SMAD to SNAI1, combining this edge with the subgraph in Fig. 9a yields a sufficient cyclic subgraph at SNAI1. This subgraph is a crucial component of the TGF *β* feedback motif (shown in Additional file 4). Fig. 9b illustrates the subgraph from RAS to E-cadherin, which is a necessary inhibitory regulator of EMT (i.e., if E-cadherin expression is lowered, EMT is activated, as can be seen in Fig. 7). E-cadherin is downregulated if the 7 transcription factors are ON, all of which can be upregulated by RAS since RAS is sufficient for them. All the upstream signals in the first row of Fig. 11 converge on the regulation of E-cadherin through these transcriptional regulators [21]. This subgraph indicates that these transcriptional regulators, and consequently EMT, can all be activated by upregulation of RAS, as was also found by dynamic simulations in [21].

**Table 3:** List of abbreviated network node names for the EMT network and the ABA network

Abbreviation	Full name (Gene name/ official name)
EMT network:	
EMT	Epithelial to Mesenchymal Transition
TGF *β*	Transforming growth factor beta
PDGF	Platelet derived growth factor
SHH	Sonic Hedgehog
SNAI1	snail homolog 1 (Drosophila)
SNAI2	snail homolog 2 (Drosophila)
ZEB1	zinc finger E-box binding homeobox 1
ZEB2	zinc finger E-box binding homeobox 2
HEY1	hairy/enhancer-of-split related with YRPW motif 1
FOXC2	forkhead box C2 (MFH-1, mesenchyme forkhead 1)
TWIST1	twist basic helix-loop-helix transcription factor 1
ERK	mitogen-activated protein kinases 1 & 3
RAS	v-Ha-ras Harvey rat sarcoma viral oncogene
	homolog
miR200	microRNA 200b
RKIP	phosphatidylethanolamine-binding protein 4;
	RAF1 the inhibitory protein
MEK	mitogen-activated protein kinase kinases 1 & 2
CHD1L	chromodomain helicase DNA binding protein 1-like
IGF1	Insulin-like growth factor 1
EGF	epidermal growth factor
HGF	hepatocyte growth factor
FGF	fibroblast growth factor 2
Wnt	wingless-type MMTV integration site family,
	member 1
NF *κ*B	nuclear factor of kappa light polypeptide gene
	enhancer in B-cells 1
*β*TrCP	beta-transducin repeat containing E3 ubiquitin
	protein ligase
H1F1 *α*	hypoxia inducible factor 1, alpha subunit
	(basic helix-loop-helix transcription factor)
FGFR	fibroblast growth factor receptor 1
PDGFR	platelet-derived growth factor receptor, alpha
	& beta polypeptides
SOS/GRB2	son of sevenless homolog 1 (Drosophila) and
	growth factor receptor-bound protein 2
GSK3 *β*	glycogen synthase kinase 3 beta
Dest_complex	Destruction complex
*β*-catenin_nuc	nuclear *β*-catenin (cadherin-associated protein)
*β*-catenin_memb	membrane-bound *β*-catenin
	(cadherin-associated protein)
GLI	GLI family zinc finger 1 & 2
DSH	dishevelled, dsh homolog 1
SUFU	suppressor of fused homolog (Drosophila)
NOTCH_ic	NOTCH (Drosophila) Homolog 1
E-cadherin	cadherin 1, type 1, E-cadherin
TCF/LEF	a basic helix-loop-helix transcription factor &
	lymphoid enhancer-binding factor 1
RAF	v-raf-1 murine leukemia viral oncogene homolog 1
ILK	integrin-linked kinase
AKT	v-akt murine thymoma viral oncogene
EGR1	early growth response 1
c-fos	FBJ murine osteosarcoma viral oncogene homolog
CsI	recombination signal binding protein for
	immunoglobulin kappa J region
ABA network:	
ABA	Abscisic acid
*p* *H*_*c*_	Increase of the cytosolic pH level
AtRAC1	small GTPase RAC1
ABI1	ABA (abscisic acid)-insensitive 1
ABI2	ABA (abscisic acid)-insensitive 2
$Ca^{2+}_{c}$	Cytosolic calcium
*C* *a* ^2+^ ATPase	Ca2+ ATPases and Ca2+/H+ antiporters responsible
	for Ca2+ efflux from the cytosol
PLD *δ*	Phospholipase D *δ*
ROS	Reactive oxygen species
RBOH	NADPH oxidases AtRBOH D and F
PA	Phosphatidic acid
PI3P5K	Phosphatidylinositol 3-phosphate 5-kinase
PtdIns(3,5)P2	Phosphatidylinositol 3,5-bisphosphate
V-PPase	vacuolar proton pyrophosphatase
RCARs	Regulatory Components of ABA Receptor
OST1	protein kinase OPEN STOMATA 1
CaIM	*C* *a* ^2+^ influx across the plasma membrane
CIS	*C* *a* ^2+^ influx to the cytosol from intracellular stores
AnionEM	Anion efflux through the plasma membrane
*K* ^+^ Efflux	*K* ^+^ efflux through the plasma membrane
*H*_2_ *O* Efflux	water efflux through the plasma membrane
MPK 9/12	Mitogen-activated protein kinases 9 and 12
CPK 3/21	Calcium-dependent protein kinases 3 and 21
SLAC1	Slow Anion Channel- associated 1

Since it is only the driver nodes that can stabilize a stable motif, we identified logic subgraphs from the network’s signals (sources) to the motifs’ driver nodes. We also look for subgraphs through which stable motifs can stabilize each other, and for subgraphs from any node in the motifs to the output node (EMT). We find that all of these subgraphs signify sufficient relationships. For example, all the stable motifs converge on the regulation of E-cadherin, and thus of the output node EMT, through the seven transcriptional regulators [21].

**Fig. 10:**
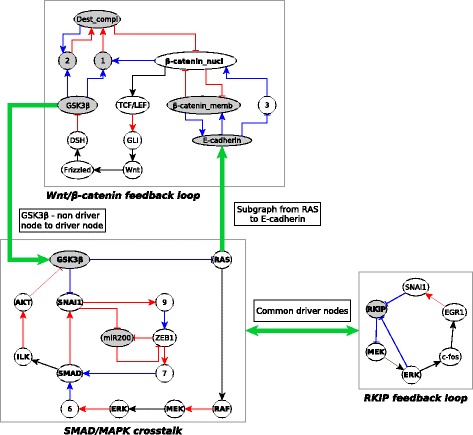
Different kinds of relationships between the stable motifs of the EMT network. The Wnt/ *β*-catenin feedback loop (top) can stabilize the SMAD/MAPK crosstalk motif (bottom left) since it fixes the state of the node GSK3 *β* which is a driver node of the SMAD/MAPK crosstalk motif. This crosstalk motif can in turn stabilize the Wnt/ *β*-catenin feedback loop motif since they share the GSK3 *β* node and there is a subgraph from one of its nodes, RAS, to E-cadherin, a member of the Wnt/ *β*-catenin feedback loop (see Fig 9b). The RAS → E-cadherin sufficient inhibitory subgraph is detailed in Fig 9b. The SMAD/MAPK crosstalk motif and the RKIP feedback loop (bottom right) can stabilize each other since they share the driver nodes MEK and ERK. Nodes with white background are in the ON state, while those with gray background are in the OFF state in the stabilized state of the motif. The names of the driver nodes are shown in boldface

Figure 10 illustrates different ways by which motifs can stabilize each other. As the Wnt/ *β*-catenin feedback loop is stabilized, each of the nodes in this motif stabilize to its corresponding fixed state, denoted by the background color of the node (white for ON, grey for OFF). The node GSK3 *β* stabilizes in the OFF state. The SMAD/MAPK crosstalk motif is essentially a necessary cyclic subgraph in which GSK3 *β* is the starting and the ending node, that is, GSK3 *β*=0 is a driver node of this motif. Thus, the stabilization of the Wnt/ *β*-catenin feedback loop leads to the stabilization of the SMAD/MAPK crosstalk motif. The RKIP feedback loop and SMAD/MAPK crosstalk have two common driver nodes: MEK and ERK. Stabilization of either of the motifs implies the stabilization of these two nodes and hence the stabilization of the other motif. This is denoted by the double arrow between these two motifs. The Wnt/ *β*-catenin motif has only one driver node - *β*-catenin nucleus which has three regulators, Destruction Complex, *β*-catenin membrane and the mediator node 3. To stabilize *β*-catenin nucleus to the ON state (which is its state corresponding to the stabilized motif), the regulators must be fixed to their corresponding states in the motif, as denoted by the node background color in the top panel of Fig. 10. When the motif SMAD/MAPK crosstalk stabilizes, the node GSK3 *β* is fixed to the OFF state and the necessary subgraph formed by GSK3 *β*, 1, 2, Destruction Complex ensures that one of the regulators of *β*-catenin nucleus is stabilized to the corresponding fixed state of the motif. Stabilization of the SMAD/MAPK motif fixes the node RAS to the ON state and the sufficient inhibitory subgraph from RAS to E-cadherin (Fig 9b) ensures that E-cadherin is stabilized to the OFF state. This stabilization of E-cadherin fixes *β*-catenin membrane to the OFF state (E-cadherin is necessary for *β*-catenin membrane) and node 3 to the ON state (E-cadherin is a necessary inhibitor of node 3). This way, stabilization of the SMAD/MAPK crosstalk motif fixes all the regulators of *β*-catenin nucleus to their stable states, hence stabilizing the *β*-catenin nucleus node itself to the ON state. Since *β*-catenin nucleus is the driver node of the Wnt/ *β*-catenin feedback loop, we can conclude that the SMAD/MAPK crosstalk motif is sufficient to stabilize the Wnt/ *β*-catenin feedback loop.

**Fig. 11:**
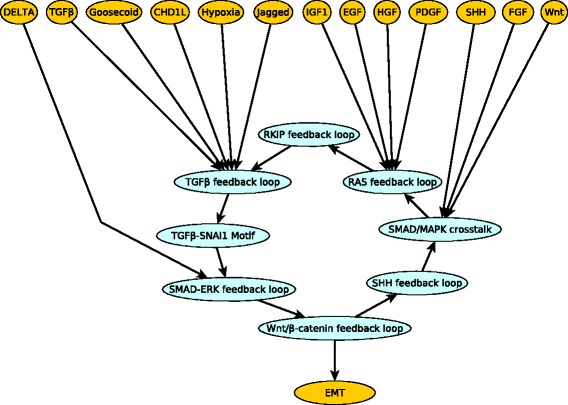
The logic backbone structure of the EMT network. All signals and the output node EMT are in yellow while the motifs are in blue. All edges represent sufficient logic relationships. An edge between a signal and a stable motif means that activating the signal can stabilize the motif. An edge between two motifs means that stabilizing one stabilizes the other. An edge from a stable motif to the output (EMT) means that the stable motif can activate the output

We represented the subgraphs starting from signals or stable motifs and ending in stable motifs or the output node as edges of the logic backbone network. All of these edges are of the same type (sufficient), thus for simplicity we do not separately label them. We find that the logic backbone contains a path from each of the sources to each of the stable motifs and to the output. Also, there is an edge or a path from each of the stable motifs to the output. Thus this logic backbone is an orientation of a complete graph. For clarity, we apply *l*-BTR to the backbone network prior to presenting it in Fig. 11. As all the edges of the logic backbone are sufficient, upregulation of any one of the signals or stabilizing any one of the motifs leads to the output node EMT=ON (mesenchymal state). This suggests that the EMT transition is a very robust outcome that can be brought on by external as well as internal drivers.

If the system is in the epithelial state, there are multiple ways to reach the mesenchymal state, e.g., through the presence of a signal or through a stabilized motif. For example, even when all the signals are absent from the system, it may reach the mesenchymal state if a random mutation or dysregulation stabilizes the state of a certain node in the opposite of its epithelial state. Interestingly, almost half of the 56 non-signal nodes are driver nodes. Hence the probability for a random node mutation leading to the mesenchymal state is *p*=27/56=0.48. Also, the probability that the effects of a random mutation die out without stabilizing a motif is given by *p* ^′^=1−*p*=0.52. The driver nodes of the EMT network are shown in Additional file 5. Many of these nodes are observed to be dysregulated in cancer patients (i.e. they are constitutively active or otherwise fixed in the functional state corresponding to the mesenchymal state of the node) [29 *–* 41]. The details of these literature evidences are specified in Additional file 6. This is consistent with our finding that the stabilized mesenchymal state of these nodes can lead to EMT, the first step toward cancer metastasis.

### Application II: Logic subgraphs and logic backbone of the ABA network

Stomatal pores, responsible for the intake of carbon dioxide and the inevitable water loss through transpiration, are crucial for maintaining the water level in a plant. The closing and opening of stomata is regulated by the turgidity of the guard cells that surround the pores; this turgidity is controlled by signaling networks that respond to multiple environmental factors such as light, carbon dioxide and humidity. In drought conditions, plants synthesize the phytohormone abscisic acid (ABA), which leads to the closure of stomata to prevent further water loss. The ABA signaling process has been studied by extensive experimental investigation and also by modeling analysis [42 *–* 45]. In particular, a recent Boolean model of ABA induced closure identifies the stable motifs of the ABA network and identifies the interventions and system trajectories that lead to the attractor associated with stomatal closure [46].

**Fig. 12:**
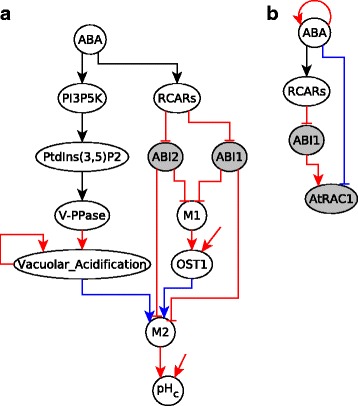
Interesting logic subgraphs in the ABA network. **a** Sufficient subgraph from ABA to pH_c_. The necessary (or sufficient inhibitory) regulators Vacuolar Acidification, ABI1, OST1 and ABI2 are together sufficient for pH_c_. The source node ABA is sufficient for Vacuolar Acidification and for OST1 while it is a sufficient inhibitor of ABI1 and for ABI2. **b** ABA has a sufficient inhibitory subgraph to AtRAC1. Along with the necessary inhibitory edge from ABA to AtRAC1, ABA is a sufficient inhibitor of the sufficient regulator of AtRAC1, ABI1. All necessary regulators are together sufficient and all sufficient regulators are together necessary unless otherwise specified via an additional edge without a starting point

**Fig. 13:**
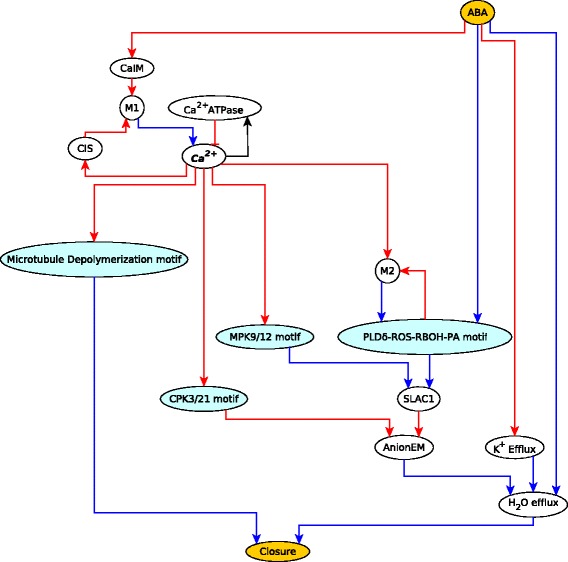
The logic backbone of the ABA network. The backbone structure contains the signal ABA, the four stable motifs, the external driver node (*C* *a* ^2+^, marked in bold and italic) of the stable motifs, the output node Closure and its key regulators. The edges are representative of logic paths or subgraphs in the Boolean model. The signal ABA and the output node Closure have yellow background while the motifs are in blue background. Multiple necessary regulators incident on the same node are together sufficient and multiple sufficient regulators incident on the same node are together necessary (i.e. their simultaneous inactivity leads to the inactivation of the target node)

We used this latter model (i.e., the same Boolean regulatory functions and assumed states for unregulated nodes) to find the logic subgraphs and logic backbone structure of the ABA induced closure network. Two interesting logic subgraphs found in the analysis are illustrated in Fig. 12. The sufficient subgraph in Fig. 12a shows that in the sustained presence of ABA, the node pH_c_ will also stabilize in the ON state. The target node pH_c_ has multiple regulators, including Vacuolar Acidification, ABI1, ABI2 and OST1, which together regulate the mediator node M2 which in turn is sufficient for pH_c_. The signal ABA is sufficient for the node Vacuolar Acidification via a path. ABA is a sufficient inhibitor of ABI1 and ABI2 through RCARs. ABA is sufficient for OST1 through the sufficient subgraph that includes ABA, RCARs, ABI1, ABI2, M1, OST1. According to the subgraph chain function detailed in Table 2, the path (or subgraph) from ABA to each of these regulators and the edge from the corresponding regulator to node M2 can be chained and give the same outcome, sufficient. Hence, ABA is sufficient for node M2, which is a sufficient regulator of pH_c_. In Fig. 12b the target node AtRAC1 has two regulators - ABI1 is sufficient while ABA is a necessary inhibitor. The signal ABA is a sufficient inhibitor of ABI1. The assumed sustained presence of ABA is equivalent to a sufficient loop on ABA. Both the sufficient inhibitory + sufficient and sufficient + necessary inhibitory pairings lead to sufficient inhibitory as given in the subgraph chain function in Table 2. Our causal analysis recovers the same four stable motifs associated to ABA induced stomatal closure as the previous analysis. The logic backbone of the ABA induced closure network focusing on the case of sustained ABA signal is presented in Fig. 13. In addition to the signal ABA and the four stable motifs, this logic backbone also highlights the node describing the cytosolic Ca^2+^ level. An interesting feature of this network is the feedback regulation of Ca^2+^ (including positive feedback through CIS and negative feedback through Ca^2+^ATPase), which yields transient increases (oscillations) in $\text {Ca}^{2+}_{\text {c}}$ in the presence of ABA. Nevertheless, even a single transient increase of $\text {Ca}^{2+}_{\text {c}}$ can stabilize three of the motifs independently and contribute, together with ABA, to the stabilization of the PLD *δ*-ROS-RBOH-PA motif. ABA is a sufficient regulator of one of the processes that generates cytosolic Ca^2+^, CaIM. The stable motifs and ABA regulate effector nodes corresponding to ion and water flow, which lead to stomatal closure (see Additional file 7).

## Discussion

In this work, we presented a representation method that incorporates all the information conveyed by Boolean regulatory functions and can serve as an alternative when full Boolean regulatory functions are not available. In this representation framework, we showed how to distill a rather complex subnetwork into a more direct causal relationship. We identified connectivity patterns that are allowed and not allowed by the logic of the relationships. An example of a pattern that is not allowed is having two independent direct regulators of a node, one of which is sufficient and the other necessary. In general, two co-pointing subgraphs, one sufficient and the other necessary for the same target node, must intersect and the source node of either of the subgraphs must lie in the intersection. We devised an algorithm to chain consecutive edges with different logical implications and search for paths and subgraphs. By testing the algorithm’s efficiency on an ensemble of random Boolean networks, we found that it should work reasonably well on large and dense Boolean networks. Further, we learnt that the algorithm is suited to work for biological networks. For example, on networks with 80 nodes and average in-degree 1.75, the observed average runtime for the algorithm was 500 ms. In contrast, the ABA network with the same size and in-degree, could be analyzed much faster – the runtime for the most complex subgraph was 85 ms. This highlights the fact that real biological networks usually have simpler and/or more canalizing logic rules than rules selected at random (which can include XOR type of rules which essentially never happen in biological systems).

We explored subgraphs that intersect or form cycles, and established the correspondence between cyclic subgraphs and previously introduced stable motifs. Identification of stable motifs must follow an exhaustive method (as it is based on determining and then filtering all cycles in an expanded network that incorporates the Boolean regulatory functions). In contrast, by searching for certain types of cyclic subgraphs, one can separately look for single driver and collective driver motifs. Motifs that can be driven by a single node stabilize more easily (e.g. by the mutation of a single node). In addition, searching for motifs driven by one node using the causal logic framework has half the space complexity compared to using the expanded network method. The driver nodes defined in this work also form a control set of nodes that can alter the system’s trajectory.

To exemplify the application of causal logic analysis to biological systems, we constructed a succinct backbone of two signal transduction networks. The logic backbone of the EMT network highlights the robustness of the EMT outcome. It shows that the presence of any one of the signals can lead to the stabilization of at least one stable motif, which then, due to the causal inter-connectivity of the motifs, leads to the stabilization of all motifs and finally to the EMT node stabilizing to the ON state. Various subgraphs in this network illustrate the strong causal effect of a node on another, faraway node. For example, the node RAS can indirectly control all the regulators of E-cadherin, as shown in Fig. 9b.

Similarly, the backbone of the ABA network is highly insightful as it illustrates the importance of feedback mechanisms in regulating the ion flow processes (Anion efflux and *K* ^+^ efflux) that are the main effectors in ABA induced stomatal closure. The node denoting the cytosolic Ca^2+^ level participates in multiple feedback loops, both positive and negative, and is an external driver node of three stable motifs. Due to negative feedback regulation, Ca^2+^ has a transient, potentially oscillating behavior in the presence of ABA. Nevertheless, the motifs, once reaching their corresponding stable states (e.g. in response to an increase in Ca^2+^), are fixed in these stable states despite the oscillating nature of Ca^2+^. The stable motifs are sufficient for anion flow and the depolarization of microtubules, which, coupled with ABA-driven *K* ^+^ efflux, are sufficient for stomatal closure. A possible follow-up to the causal logic analysis presented here is to consider the cases wherein constitutive activity of certain network elements can lead to stomatal closure even in the absence of ABA. The model of [46] can reproduce most of these results by assuming an additional feedback. Constructing the backbone structure corresponding for the interventions that can lead to closure, with or without the newly predicted interaction, may lead to additional insights and predictions.

Here we focused on the logic implication of a sustained state of a source node on a target node. While this is the most frequently encountered example, the interplay between the complex dynamics and functional importance of Ca^2+^ in the ABA network suggests that elucidating the causal implications of oscillatory states is an interesting topic for future theoretical exploration. We propose that one can attribute a logic implication to an edge in the non-stationary case as well if the time delay introduced by the edge (i.e. the time difference between the state change of the regulator and the state change of the target) is sufficiently short compared to the time scale of the changes in the source node’s state. The identification of the constraint on the time delays that makes the identification of logic relationships possible is a topic of future work.

## Conclusion

We have presented a condensation technique for Boolean networks, which ultimately yields a logic backbone that expresses the relationship between external/internal signals and output nodes in an easy to understand manner. The causal logic framework can also be used for other network studies. In this work, we started from known Boolean regulatory functions to obtain the logic properties of edges. One can also do the converse, use the experimental information on causal relationships to infer the Boolean regulatory functions consistent with them. In cases when there is insufficient information to infer unique Boolean functions, we can still conclude certain relationships. For example, as knockout experiments are more prevalently conducted than constitutive activation experiments, we are more likely to know all necessary regulators than all sufficient regulators of a certain node. Even if not all sufficient regulators are known, all the conclusions on linear paths and most sufficient subgraphs (and hence stable motifs formed by the sufficient cyclic subgraphs) would still be valid. Furthermore, we can also use the knowledge of subgraphs to fit newly-emerging evidence into the network structure. For example, consider that we experimentally know that a source node is sufficient for a target node (in the presence of all the molecules necessary for life), and this is realized via a subgraph in the network. If a new necessary regulator of this target node is discovered, we can use our result on co-pointing subgraphs to infer that the source is sufficient for this new regulator.

## Additional files

Additional file 1 Pseudocode and runtime of the algorithm to find logic subgraphs. (PDF 332 kb)

Additional file 2 The proofs of the propositions regarding the properties of logic subgraphs. (PDF 82 kb)

Additional file 3 Explanation of extended cyclic subgraphs with collective driver nodes. (PDF 79 kb)

Additional file 4 Supplementary Figure 1, containing all eight motifs of the EMT network in causal logic representation. (DOCX 115 kb)

Additional file 5 Supplementary Table 1, containing the list of all nodes of the network and their states in the epithelial and mesenchymal states. (DOCX 6 kb)

Additional file 6 Literature evidence in support of the driver nodes of the EMT network: their dysregulation frequently occurs in and may be causative of cancer. (PDF 67 kb)

Additional file 7 Stable motifs and node states of the ABA network nodes corresponding to the closure attractor. (DOCX 24 kb)

## Abbreviations

BTR: Binary transitive reduction
PVC: Pseudo vertex collapse
*l*-BTR: logical binary transitive reduction
LVC: logical vertex collapse
