# Supplementary material for: A framework to find the logic backbone of a biological network
Source: BMC Syst Biol. 2017 Dec 6;11:122. doi: 10.1186/s12918-017-0482-5 (PMC5719532; doi:10.1186/s12918-017-0482-5)
Supplement: Supplementary file 1 — Pseudocode and runtime of the algorithm to find logic subgraphs. (PDF 332 kb) [file 12918_2017_482_MOESM1_ESM.pdf]

Pseudocode and runtime of the algorithm to find logic subgraphs

The programs used to construct a causal logic network from Boolean rules, analyze and reduce the network, and find subgraphs and motifs can be found in the github repository `https://github.com/parulm/suff_necc`. The way Boolean rules can be translated to edge properties is detailed in Figure 1 of the main text. Network analysis and reduction frequently uses the path and subgraph chain functions explained in Tables 1 & 2 in the main text. The subgraph finding function looks for a subnetwork (set of paths) with logical implication from the specified source node to the specified target node. For example, there can be a sufficient subgraph from a source to a target even if the target has necessary regulators only; this is possible if the source is sufficient for each of the regulators. The source may be sufficient for the regulators via a path or a subgraph. In this case, when the subgraph finding function finds a node with necessary regulators, it makes a recursive call to itself to find subgraphs/paths from the source node to each of the regulators.
The pseudo code detailed below was used to find a subgraph between nodes `source` and `target`. This function is executed for all paths one by one between the two nodes. To find cyclic subgraphs, that is, stable motifs, this function is run for each node with `target=source`.

```
find_subgraph(source, target):
 for path in all_paths_from_source_to_target:
  #See if there is a path with logic implication.
  if type_of_path is not None:
   return type_of_path
  else:
   #There is no direct path, so we'll look for a subgraph.
   #Set prev_edge_type to suff & necc since this is the identity element of
   #path chaining function.
   prev_edge_type <- suff/necc
   #Scan the path node by node.
   for node in path:
    new_edge_type <- edge type of node to next_node in path
    #Variable rel is set to chain function (see Table 1 in the main text) with
```

```
    #prev_edge_type and new_edge_type as arguments.
    rel <- path_chain(prev_edge_type, new_edge_type)
    #Initializing variable srel which denotes the possible subgraph type
    srel <- None
    if rel = None:
     #This would be true when chaining of prev_edge_type and new_edge_type
     #does not give a particular logic implication, i.e., returns '-' in Table 1
     #of main text. For example, chaining sufficient and necessary.
     #Setting srel to possible subgraph type as per the subgraph chain function
     #(see Table 2 in the main text).
     srel <- subgraph_chain(prev_edge_type, new_edge_type)
     #Once we have a different type of edge(for example, necessary), we check if
     #the source matches (i.e., source is sufficient for all the necessary regulators).
     for all regulators of next_node in path:
      #Set ptype to the logic implication of source on the regulator.
      ptype <- find_subgraph(source, regulator, path from source to regulator)
      #The possible subgraph type for all regulators must be srel.
      if subgraph_chain(ptype, edge_type b/w regulator and next_node) = srel:
       continue
      else:
       srel <- None
     if srel is not None:
      rel <- srel
    prev_edge_type <- new_edge_type
    if rel is None:
     #Stop scanning this path
     break
   if rel is None:
    #Proceed to the next path
    continue
return rel
```

The flowchart below illustrates the algorithm with an example, which is the sufficient subgraph AHFBGDCNMELK from Figure 4 of main text. The general algorithm is detailed on the left while the particular execution on AK subgraph is on the right. The abbreviations used for causal logic implications are as follows: s-sufficient; n-necessary; si-sufficient inhibitory.

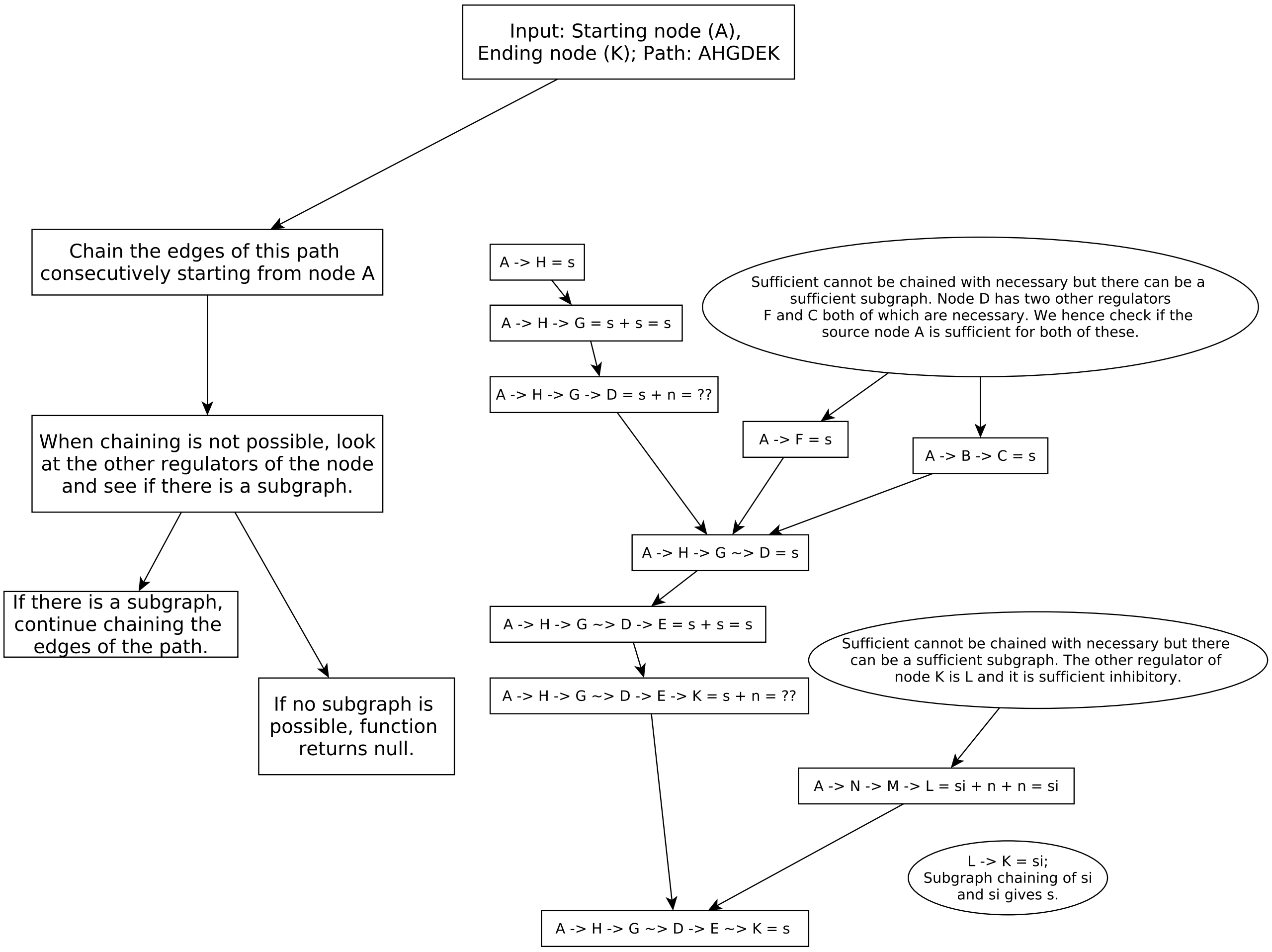

Runtime complexity of the algorithm

To study the complexity of this algorithm, we identified the stable motifs (cyclic subgraphs) in an ensemble of random Boolean networks. Except for some rare cases of self-loops and simple cycles, cyclic subgraphs are typically more complex than simple logic subgraphs thus their identification is representative of the "worse-case" runtime of this algorithm. In order to obtain a random Boolean network, we constructed a random graph with given number of nodes and given average in-degree using a built-in function in networkx. We then assigned a random logic function for each of the nodes of this network. We analyzed random Boolean networks with 20-60 nodes and in-degree varying between 1.2-2.1. In general, for networks with given number of nodes, the algorithm's runtime increased exponentially with the average in-degree of the network. Two of the runtime complexity plots are illustrated in Figures S1 and S2.

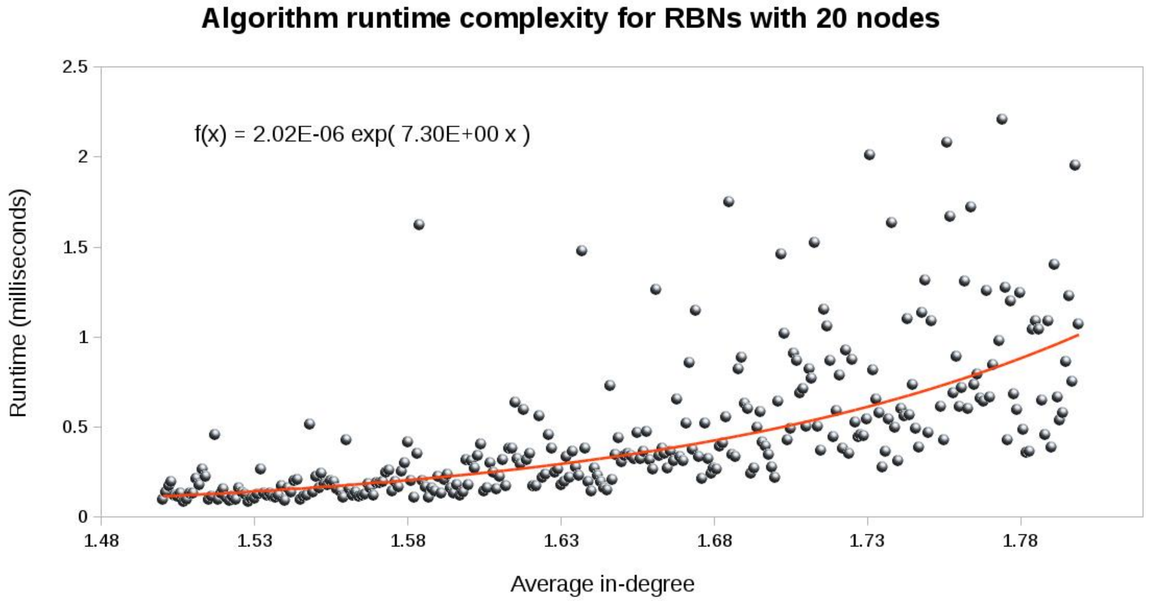

Figure S1: Algorithm runtime complexity for an ensemble of random Boolean networks with 20 nodes. X-axis represents average in-degree of the network while the Y-axis represents the average runtime to find a cyclic subgraph, in milliseconds. Each data point (pearl symbol) is an averaging over 100 RBNs and each RBN has approximately 3-4 cyclic subgraphs. The exponential fit equation is shown in top left.

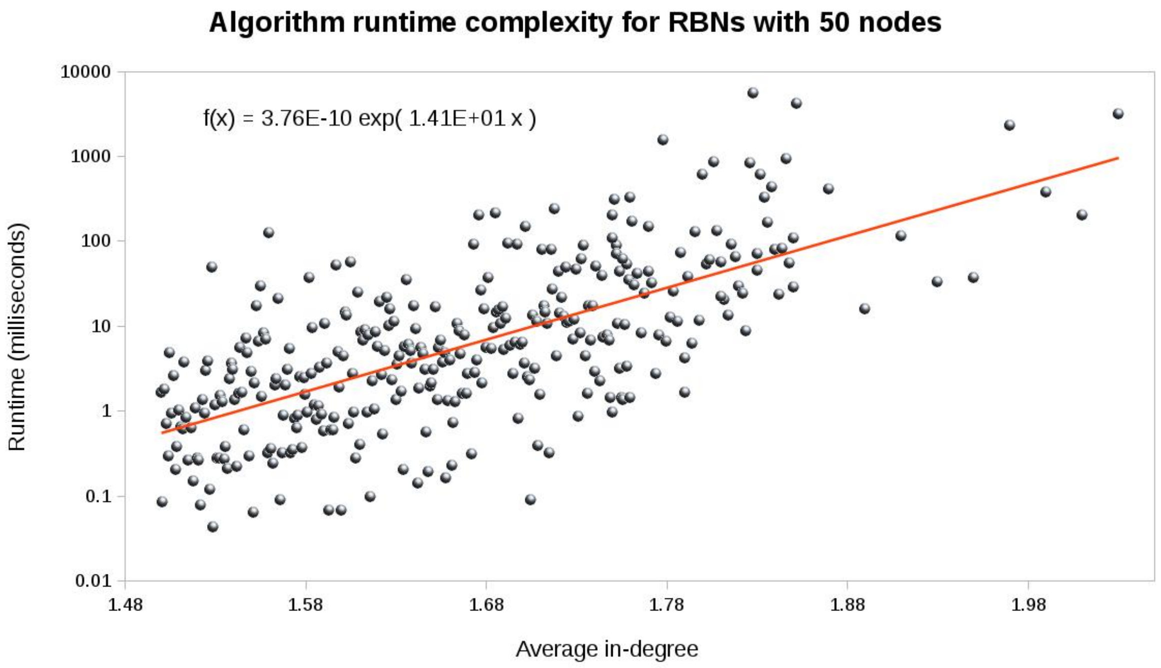

Figure S2: Algorithm runtime complexity for an ensemble of random Boolean networks with 50 nodes. X-axis represents average in-degree of the network while the Y-axis, on logarithmic scale, represents the average runtime to find a cyclic subgraph, in milliseconds. Each data point is average over 10 RBNs and each RBN has approximately 7-10 cyclic subgraphs. The log scale data is fitted to a linear plot and the equation in top left shows the exponential functional form of the linear fit.
