# Supplementary material for: A framework to find the logic backbone of a biological network
Source: BMC Syst Biol. 2017 Dec 6;11:122. doi: 10.1186/s12918-017-0482-5 (PMC5719532; doi:10.1186/s12918-017-0482-5)
Supplement: Supplementary file 2 — The proofs of the propositions regarding the properties of logic subgraphs. (PDF 82 kb) [file 12918_2017_482_MOESM2_ESM.pdf]

Proofs for Section 3.2: Properties of Logic Subgraphs

<u>*Proposition:*</u> *A sufficient subgraph that does not contain any inhibitory edges cannot intersect a necessary cycle.*

<u>Proof:</u> Let S_s be a sufficient subgraph and C_n be a necessary cycle. [Here a cycle means a closed path and not a cyclic subgraph].
$I = S_s \cap C_n = \{\text{all nodes and edges in the intersection}\}$
Since C_n is a necessary cycle, all edges in it are necessary.
Let e be an edge, $\forall e \in I$, e is necessary. Consider $e = A \to B$, then, $A, B \in I \Rightarrow A, B \in S_s$. In a sufficient subgraph (without inhibitory edges), the starting node must be sufficient for all the necessary regulators of any node (if it has necessary regulators). Hence, $start\_node(S_s)$ is sufficient $\forall node \in predecessor\_set(B)$.
Since $B \in C_n$, there exists a predecessor B' of B such that $B' \in C_n$ and the $start\_node(S_s)$ is sufficient for B, hence, $B' \in S_s$.
Now, there is a predecessor B'' of B' such that $B'' \in C_n$; $start\_node(S_s)$ must also be sufficient for B'' [since it is sufficient for all predecessors of B']. Hence, we have $B'' \in S_s$. Iterating this argument shows that all nodes of C_n must lie in S_s. Now, if $C_n \subset S_s$, every node in C_n is a necessary regulator of some other node in C_n. Hence, $start\_node(S_s)$ must be sufficient for every node of C_n, which would in turn depend on its being sufficient for another node [Since $start\_node(S_s)$ cannot be directly sufficient to any of these nodes via an edge since such a combination of relationships is not possible]. We, hence, get into an infinite loop, which in turn prevents S_s from being a sufficient subgraph, which is a contradiction.

<u>*Proposition:*</u> *A sufficient subgraph that contains inhibitory edges can intersect a necessary subgraph only if the intersection follows a sufficient inhibitory section.*

<u>Proof:</u> Let S_s be a sufficient subgraph and C_n be a necessary cycle [Again, a cycle here is a closed path and not a cyclic subgraph].
$I = S_s \cap C_n = \{\text{all nodes and edges in the intersection}\}$.
If $start\_node(S_s)$ is fixed in the ON state, then all nodes in I would stabilize to a fixed state [It must be the same fixed state for all nodes: even if just one of them is OFF, the

rest would also stabilize to OFF as all nodes in I are a part of necessary cycle C_n]. If that fixed state is ON, then $start\_node(S_s)$ must be sufficient for each node of C_n which is not possible [as sketched in the above proof]. Hence that fixed state is OFF.
Pick any node $n \in I$. As $start\_node(S_s)$ is fixed at ON, n would go to the OFF state implying that there must be a sufficient inhibitory section from $start\_node(S_s)$ to n.

Proposition: *If there are two co-pointing subgraphs such that one is necessary while the other is sufficient and the source node of the sufficient subgraph is a signal node (i.e. zero in-degree), then*

1. *they must intersect*
2. *the starting node of the necessary subgraph must lie in the intersection*
3. *the starting node of the sufficient subgraph is sufficient for the starting node of the necessary subgraph.*

Proof: Let S_s be the sufficient subgraph and S_n be the necessary subgraph. Since these are co-pointing subgraphs, they have the same ending node, i.e., $end\_node(S_s) = end\_node(S_n) = T$.

1. Consider the last edge of the S_s [the choice does not matter, we can alternatively consider the last edge of S_n] which is incident on T.
   Case I: This is a sufficient edge $\Rightarrow$ other regulators of T are either necessary inhibitory or sufficient. No sort of subgraph or path followed by a necessary inhibitory or sufficient edge would yield a necessary subgraph [look at the columns corresponding to sufficient or necessary inhibitory in the chain function in Table 1: none of these contain necessary]. Hence, for S_n to exist in this scenario, there must be an intersection of S_n and S_s and that intersection must contain at least one of the regulators of T.
   Case II: The last edge is necessary. For S_s (a sufficient subgraph) to end in a necessary edge, $start\_node(S_s)$ must be sufficient (sufficient inhibitory) for all other necessary (sufficient inhibitory) regulators of T. Hence S_s contains all the regulators of T. Since S_n must go via at least one of the regulators of T [there is no other way to complete a subgraph to T], there exists an intersection of S_n and S_s.

2. $I = S_s \cap S_n$. Assume that $start\_node(S_n) \notin I$.
   By definition, a sufficient subgraph is a subgraph for which, if the starting node is set to ON, the ending node stabilizes to ON, and the nodes contained in the subgraph go to a fixed state, irrespective of the state of the rest of the network. If $start\_node(S_n)$ is strictly outside of S_s, and $start\_node(S_s)$ is set to ON, then T must stabilize to ON irrespective of the state of $start\_node(S_n)$. This violates the statement that $start\_node(S_n)$ is necessary for T. Hence $start\_node(S_n) \in I$.

3. Now that we have established that $start\_node(S_n) \in S_s$, fixing $start\_node(S_s)$ to ON would also stabilize the state of $start\_node(S_n)$ to a fixed state [since it stabilizes the states of all the nodes in the subgraph]. Since $start\_node(S_n)$ is necessary for T, this must be the ON state. Hence, we have that $start\_node(S_s) = ON \Rightarrow start\_node(S_n) = ON$, implying that $start\_node(S_s)$ is sufficient for $start\_node(S_n)$.
