# Supplementary material for: A framework to find the logic backbone of a biological network
Source: BMC Syst Biol. 2017 Dec 6;11:122. doi: 10.1186/s12918-017-0482-5 (PMC5719532; doi:10.1186/s12918-017-0482-5)
Supplement: Supplementary file 3 — Explanation of extended cyclic subgraphs with collective driver nodes. (PDF 79 kb) [file 12918_2017_482_MOESM3_ESM.pdf]

Extended cyclic subgraphs with collective driver nodes

There can be logic subgraphs from multiple nodes to multiple nodes, wherein fixing the state of all the starting nodes fixes the states of all the ending nodes. An example of such a subgraph is shown in Figure 1.

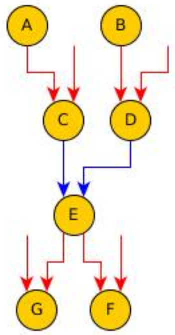

Figure 1: A basic example of an extended logic subgraph. There is a sufficient subgraph from $\{A, B\}$ to $\{G, F\}$, i.e., if nodes A and B are set to ON, then nodes G and F would also stabilize to the ON state.

The algorithm detailed in Additional File 1 was adapted to search for extended subgraphs: the recursive step where the `nested_subgraph` function is called is executed for all possible sources. The entire function is re-run for every target node.
If such a subgraph is cyclic (i.e. the same set of nodes is both the beginning and end of the subgraph), it determines a stable motif with more than one driver node. A good example of an extended cyclic subgraph is the SMAD-ERK feedback loop in Figure 8 of the main text. There is a sufficient subgraph from SMAD to 7; SNAI1 and ERK together stabilize RKIP to OFF which fixes MEK to ON, hence there is a sufficient extended subgraph from {ERK, SMAD} to MEK. Since nodes 6 and 7 together stabilize SMAD to ON and MEK is the only regulator of ERK, the two nodes (SMAD and ERK) are sufficient for themselves, hence forming a cyclic subgraph with collective driver nodes.
Theoretically, the set of all the cyclic subgraphs with any number of driver nodes is equivalent to the set of stable motifs as defined in [1].

References

[1] J. G. Zañudo and R. Albert, "An effective network reduction approach to find the dynamical repertoire of discrete dynamic networks," *Chaos: An Interdisciplinary Journal of Nonlinear Science*, vol. 23, no. 2, p. 025111, 2013.
