# Supplementary material for: A framework to find the logic backbone of a biological network
Source: BMC Syst Biol. 2017 Dec 6;11:122. doi: 10.1186/s12918-017-0482-5 (PMC5719532; doi:10.1186/s12918-017-0482-5)
Supplement: Supplementary file 4 — Supplementary Figure 1, containing all eight motifs of the EMT network in causal logic representation. (DOCX 115 kb) [file 12918_2017_482_MOESM4_ESM.docx]

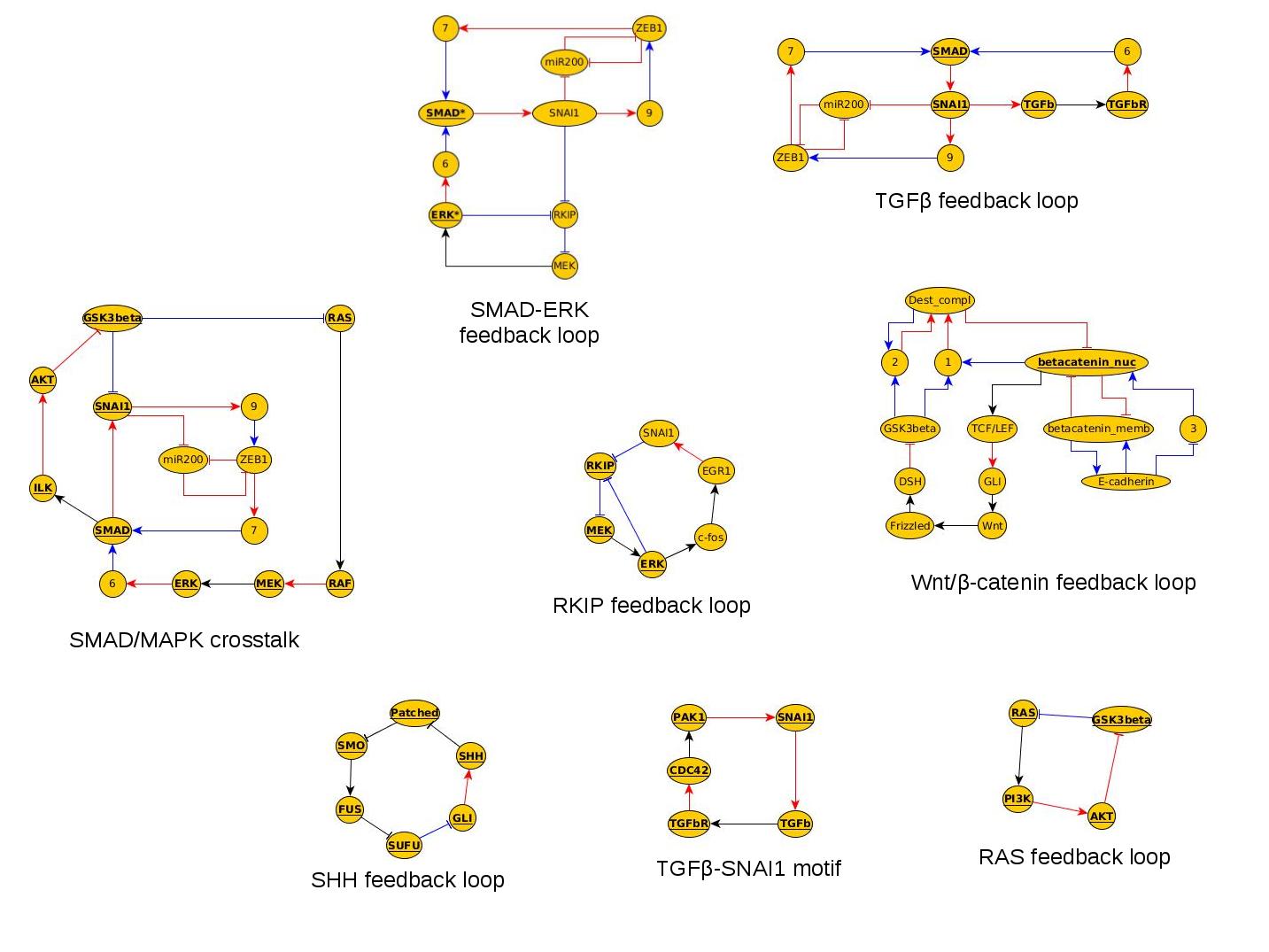


Figure S1: **Motifs of the EMT network**: The nodes with boldface and underlined labels are driver nodes (internal or external). The internal driver nodes can be easily identified as they are marked inside the motif itself. There are multiple external driver nodes, some examples are CDC42 and PAK1 for the TGFβ feedback loop; PI3K for the SMAD/MAPK crosstalk. The SMAD-ERK feedback loop has two collective driver nodes: SMAD and ERK, hence both these nodes need to fixed in the ON state to stabilize the motif. Red edges are sufficient, blue means necessary and black means sufficient and necessary.
