# Supplementary material for: A framework to find the logic backbone of a biological network
Source: BMC Syst Biol. 2017 Dec 6;11:122. doi: 10.1186/s12918-017-0482-5 (PMC5719532; doi:10.1186/s12918-017-0482-5)
Supplement: Supplementary file 5 — Supplementary Table 1, containing the list of all nodes of the network and their states in the epithelial and mesenchymal states. (DOCX 6 kb) [file 12918_2017_482_MOESM5_ESM.docx]

| Node Name | E  Table S1: **List of nodes and their states in the epithelial (E) and mesenchymal (M) states of the reduced EMT network (Figure 7 of main text)**. A gray block signifies the OFF state and white signifies the ON state. The driver nodes are written in bold. Notes: 1. Dest_compl forms a self-loop in the Epithelial state and hence it can be in any state; 2. betacatenin_memb and E-cadherin form a positive feedback loop. As a result E-cadherin can be OFF causing betacatenin_memb to be OFF and EMT to be ON while the rest of the network is in the epithelial state, resulting in a hybrid state of the system; 3. AXIN2 also forms a self-loop in the epithelial state and can hence be ON or OFF. The source nodes that represent external signals are not included in this table. All of these nodes are OFF in the epithelial state. One or more of them can be ON in the mesenchymal state (when this state is driven by external signals), or they could be OFF (if the mesenchymal state is driven by deregulation of an internal node). The nodes whose state is directly determined by the state of signal nodes (namely, PDGFR and HIF1α) are also not included. | M |
| --- | --- | --- |
| Dest_compl | 1 |  |
| **GLI** |  |  |
| **TGFb** |  |  |
| **SMAD** |  |  |
| **RKIP** |  |  |
| **NOTCH_ic** |  |  |
| **GSK3beta** |  |  |
| miR200 |  |  |
| **RAS** |  |  |
| **ERK** |  |  |
| **betacatenin_memb** | 2 |  |
| **DSH** |  |  |
| **SUFU** |  |  |
| **SNAI1** |  |  |
| ZEB1 |  |  |
| **betacatenin_nuc** |  |  |
| SNAI2 |  |  |
| betaTrCP |  |  |
| AXIN2 | 3 |  |
| E-cadherin | 2 |  |
| ZEB2 |  |  |
| NFKb |  |  |
| SOS/GRB2 |  |  |
| EMT | 2 |  |
