# Supplementary material for: A framework to find the logic backbone of a biological network
Source: BMC Syst Biol. 2017 Dec 6;11:122. doi: 10.1186/s12918-017-0482-5 (PMC5719532; doi:10.1186/s12918-017-0482-5)
Supplement: Supplementary file 6 — Literature evidence in support of the driver nodes of the EMT network: their dysregulation frequently occurs in and may be causative of cancer. (PDF 67 kb) [file 12918_2017_482_MOESM6_ESM.pdf]

Literature evidence in support of the driver nodes of the EMT network: their dysregulation frequently occurs in and may be causative of cancer

TGFβ is upregulated in 40% of patients with heptocellular carcinoma [1], [2]. A tendency towards GLI overexpression in human breast cancer has been shown [3]. The Raf kinase inhibitor protein (RKIP) is known to be a prognostic marker in prostate cancer [4], [5]. GSK3β activity is known to be dysregulated and SNAI1 activity is known to be upregulated during the development and progression of human breast cancer [6], [5]. Wnt and betacatenin_nuc upregulation has been associated with cancer cell growth and maintenance [7], [6]. Upregulation and overexpression of DVL-1, the human counterpart of the DSH gene, in primary breast cancer has been shown [8]. A significant subset of children with medulloblastoma have been reported to carry germline and somatic mutations in SUFU [9]. B-RAF gene mutations have been reported in 66% of malignant melanomas [10], over 60% of which show elevated kinase activity and signal to ERK [11]. Aberrant NOTCH signalling has been linked to tumorigenesis and cancer [12]. Experimental data has suggested SMO activity to be a major mediator of breast tumor cell growth [13]. Upregulation of Frizzled-7 in human gastric cancer has been reported [14].

References

[1] S. N. Steinway, J. G. Zañudo, W. Ding, C. B. Rountree, D. J. Feith, T. P. Loughran, and R. Albert, "Network modeling of tgfβ signaling in hepatocellular carcinoma epithelial-to-mesenchymal transition reveals joint sonic hedgehog and wnt pathway activation," *Cancer research*, vol. 74, no. 21, pp. 5963–5977, 2014.

[2] M. Abou-Shady, H. U. Baer, H. Friess, P. Berberat, A. Zimmermann, H. Graber, L. I. Gold, M. Korc, and M. W. Büchler, "Transforming growth factor betas and their signaling receptors in human hepatocellular carcinoma," *The American journal of surgery*, vol. 177, no. 3, pp. 209–215, 1999.

[3] A. ten Haaf, N. Bektas, S. von Serenyi, I. Losen, E. C. Arweiler, A. Hartmann, R. Knüchel, and E. Dahl, "Expression of the glioma-associated oncogene homolog

(gli) 1 in human breast cancer is associated with unfavourable overall survival," *BMC cancer*, vol. 9, no. 1, p. 1, 2009.

[4] Z. Fu, Y. Kitagawa, R. Shen, R. Shah, R. Mehra, D. Rhodes, P. J. Keller, A. Mizokami, R. Dunn, A. M. Chinnaiyan, *et al.*, "Metastasis suppressor gene raf kinase inhibitor protein (rkip) is a novel prognostic marker in prostate cancer," *The Prostate*, vol. 66, no. 3, pp. 248–256, 2006.

[5] S. Baritaki, S. Huerta-Yepez, A. Sahakyan, I. Karagiannides, K. Bakirtzi, A. Jazirehi, and B. Bonavida, "Mechanisms of nitric oxide-mediated inhibition of emt in cancer: inhibition of the metastasis-inducer snail and induction of the metastasis-suppressor rkip," *Cell Cycle*, vol. 9, no. 24, pp. 4931–4940, 2010.

[6] J. I. Yook, X.-Y. Li, I. Ota, C. Hu, H. S. Kim, N. H. Kim, S. Y. Cha, J. K. Ryu, Y. J. Choi, J. Kim, *et al.*, "A wnt–axin2–gsk3β cascade regulates snail1 activity in breast cancer cells," *Nature cell biology*, vol. 8, no. 12, pp. 1398–1406, 2006.

[7] T. Reya and H. Clevers, "Wnt signalling in stem cells and cancer," *Nature*, vol. 434, no. 7035, pp. 843–850, 2005.

[8] T. Nagahata, T. Shimada, A. Harada, H. Nagai, M. Onda, S. Yokoyama, T. Shiba, E. Jin, O. Kawanami, and M. Emi, "Amplification, up-regulation and over-expression of dvl-1, the human counterpart of the drosophila disheveled gene, in primary breast cancers," *Cancer science*, vol. 94, no. 6, pp. 515–518, 2003.

[9] M. D. Taylor, L. Liu, C. Raffel, C.-c. Hui, T. G. Mainprize, X. Zhang, R. Agatep, S. Chiappa, L. Gao, A. Lowrance, *et al.*, "Mutations in sufu predispose to medulloblastoma," *Nature genetics*, vol. 31, no. 3, pp. 306–310, 2002.

[10] H. Davies, G. R. Bignell, C. Cox, P. Stephens, S. Edkins, S. Clegg, J. Teague, H. Woffendin, M. J. Garnett, W. Bottomley, *et al.*, "Mutations of the braf gene in human cancer," *Nature*, vol. 417, no. 6892, pp. 949–954, 2002.

[11] P. T. Wan, M. J. Garnett, S. M. Roe, S. Lee, D. Niculescu-Duvaz, V. M. Good, C. G. Project, C. M. Jones, C. J. Marshall, C. J. Springer, *et al.*, "Mechanism of activation of the raf-erk signaling pathway by oncogenic mutations of b-raf," *Cell*, vol. 116, no. 6, pp. 855–867, 2004.

[12] E. J. Allenspach, I. Maillard, J. C. Aster, and W. S. Pear, "Notch signaling in cancer," *Cancer biology & therapy*, vol. 1, no. 5, pp. 466–476, 2002.

[13] A. Pledgie, Y. Huang, A. Hacker, Z. Zhang, P. M. Woster, N. E. Davidson, and R. A. Casero, "Spermine oxidase smo (paoh1), not n1-acetylpolyamine oxidase pao, is the primary source of cytotoxic h2o2 in polyamine analogue-treated human breast cancer cell lines," *Journal of Biological Chemistry*, vol. 280, no. 48, pp. 39843–39851, 2005.

[14] H. Kirikoshi, H. Sekihara, and M. Katoh, "Up-regulation of frizzled-7 (fzd7) in human gastric cancer," *International journal of oncology*, vol. 19, no. 1, pp. 111–115, 2001.
