# Supplementary material for: A framework to find the logic backbone of a biological network
Source: BMC Syst Biol. 2017 Dec 6;11:122. doi: 10.1186/s12918-017-0482-5 (PMC5719532; doi:10.1186/s12918-017-0482-5)
Supplement: Supplementary file 7 — Stable motifs and node states of the ABA network nodes corresponding to the closure attractor. (DOCX 24 kb) [file 12918_2017_482_MOESM7_ESM.docx]

**Stable motifs of the ABA induced closure network given in Figure 13**:


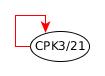


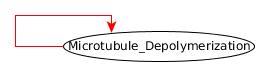


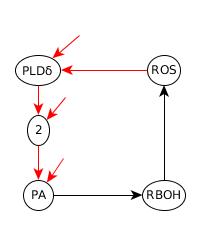


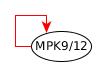


Figure S4: Stable motifs of the ABA induced closure network mentioned in Figure 13 of the main text. All the nodes are ON in the stabilized state of the motifs. Every node of each motif is a driver node, except for mediator nodes. Edges without starting points signify the existence of other regulators.

**States of the nodes that appear in Figure 13 corresponding to the closure attractor**:

ON: ABA, CaIM, CIS, SLAC1, AnionEM, K^+^ Efflux, H_2_O Efflux and Closure

oscillating: Ca^2+^ , Ca^2+^ ATPase
